# Effects of L-carnitine supplementation on lipid profile in adult patients under hemodialysis: a systematic review and meta-analysis of RCTs

**DOI:** 10.3389/fmed.2024.1454921

**Published:** 2024-12-02

**Authors:** Mehdi Karimi, Samira Pirzad, Seyed Morteza Ali Pourfaraji, Peyvand Parhizkar Roudsari, Niyousha Shirsalimi, Sajad Ahmadizad

**Affiliations:** ^1^Faculty of Medicine, Bogomolets National Medical University (NMU), Kyiv, Ukraine; ^2^Faculty of Medicine, Islamic Azad University, Tehran Medical Sciences Branch, Tehran, Iran; ^3^Faculty of Medicine, Tehran University of Medical Science (TUMS), Tehran, Iran; ^4^Faculty of Medicine, Hamadan University of Medical Science (UMSHA), Hamadan, Iran; ^5^Department of Biological Sciences in Sport, Faculty of Sport Sciences and Health, Shahid Beheshti University, Tehran, Iran

**Keywords:** L-carnitine, carnitine, serum lipid, hemodialysis, chronic kidney disease, nephropathy, meta-analysis

## Abstract

**Background:**

Chronic kidney disease (CKD) affects 10% of the global population and leads to end-stage renal disease (ESRD). Hemodialysis is a common treatment for ESRD, but patients often have low carnitine levels, leading to dyslipidemia, a risk factor for cardiovascular disease and the leading cause of mortality. This study aimed to assess the effects of L-carnitine on lipid profiles in adult hemodialysis patients.

**Methods:**

A comprehensive search was conducted across the online databases from inception to June 2024 to identify randomized clinical trials (RCTs) evaluating the effects of L-carnitine on lipid profiles in hemodialysis patients. Data extraction and quality assessment were performed, focusing on primary outcomes, including changes in triglycerides (TG), total cholesterol (TC), high-density lipoprotein (HDL), low-density lipoprotein (LDL), and very low-density lipoprotein (VLDL), and secondary outcomes including blood pressure (BP) and body mass index (BMI).

**Results:**

A total of 28 RCTs were eligible for the current systematic review, including 1,340 hemodialysis patients (671 intervention, 669 control). There were no significant differences in the mean change of TG (SMD: −0.006; 95% CI, −0.272 to 0.259; *P* = 0.95), TC (SMD: −0.086; 95% CI, −0.253 to −0.079; *P* = 0.29), HDL (SMD: 0.060; 95% CI, −0.057 to 0.177; *P* = 0.29), LDL (SMD: −0.075; 95% CI, −0.274 to 0.123; *P* = 0.43), VLDL (SMD: −0.064; 95% CI, −0.272 to 0.142; *P* = 0.51), BMI (SMD: −0.025; 95% CI, −0.139 to 0.088; *P* = 0.56), systolic BP (SMD: 0.055; 95% CI, −0.110 to 0.220; *P* = 0.43), and diastolic BP (SMD: −0.028; 95% CI, 0.156 to 0.099; *P* = 0.56). The same insignificant findings were observed after conducting a subgroup analysis based on the route of administration (intravenous *vs*. Oral).

**Conclusion:**

L-carnitine supplementation does not significantly change and improve the serum lipid profile, including TG, TC, HDL, LDL, and VLDL levels. Additionally, it has no notable effects on BMI, systolic, or diastolic BP.

## 1 Introduction

Chronic kidney disease (CKD) is a progressive loss of kidney function and a permanent clinical syndrome that is known by the kidney’s failure to filter waste products and remove excessive fluid from the body (1). CKD is a prevalent condition that affects about 10% of the global population (2). CKD can progress to end-stage renal disease (ESRD), which is a considerable cause of reduced quality of life and premature mortality (3, 4). Although the overall mortality rate among the ESRD population has been improving over time, the mortality rate remains relatively high (up to 30%) within the initial year after transitioning from CKD to ESRD (5, 6). Hemodialysis (HD) is the most prevalent type of kidney replacement therapy globally and is known as a standard therapeutic option in patients with ESRD (7). Patients undergoing hemodialysis often have dyslipidemia, a known risk factor for cardiovascular disease (CVD) and the primary cause of death among HD patients (8, 9).

L-carnitine (LC) is a naturally occurring compound that plays a role in the metabolism of fatty acids. LC transports long-chain fatty acids into the mitochondrial matrix for energy conversion through β-oxidation, enabling cells to break down fat for stored energy (10). It also depletes acyl groups from mitochondria in tissues and improves adipokine concentration, potentially improving lipid profile and preventing related diseases (11–13). Patients undergoing dialysis frequently have deficiencies in carnitine. Serum-free carnitine levels in hemodialysis patients (HD) are much lower than in the general population (14). Carnitine deficiency contributes to the development of various pathological conditions, such as cardiac dysfunction, muscle weakness, and erythropoietin-resistant anemia in patients undergoing hemodialysis (15).

Numerous randomized clinical trials (RCTs) have been conducted to evaluate the efficacy of LC on lipid profile in patients undergoing HD; however, the contradictions between the reports are evident. Among them, some studies concluded that LC has a promising effect on lipid profile (16), whereas, others did not find acceptable evidence for the relationship between LC and the improvement of dyslipidemia in HD patients (17).

Research indicates that hemodialysis patients often suffer from carnitine deficiency (18–20). However, while several studies have explored the effects of LC in healthy populations, there is a lack of evidence regarding its impact on hemodialysis patients specifically. Given these discrepancies in the literature, this study aims to evaluate the effects of LC supplementation on the lipid profiles of hemodialysis patients by analyzing data from randomized controlled trials (RCTs). This review seeks to determine whether LC can be recommended as a therapeutic approach for managing dyslipidemia and improving cardiovascular function and overall health in this population.

## 2 Methods

This investigation was conducted according to the guidelines outlined in the Preferred Reporting Items for Systematic Reviews and Meta-Analysis (PRISMA) statement (21).

The protocol for this systematic review has been registered with PROSPERO (the International Prospective Register of Systematic Reviews) under the registration number CRD42024555147.

### 2.1 Search strategy

A systematic search was conducted across PubMed, Web of Science, Scopus, and Embase databases, covering all available records from their inception to May 2024. The aim was to identify RCTs evaluating the effects of LC supplementation on the lipid profile of patients undergoing hemodialysis. The following MeSH and related search terms were used: (“L-Carnitine” OR Carnitine OR Levocarnitine OR Bicarnesine OR “Vitamin BT” OR “Acetate Free Biofiltration” AND “lipid profile” OR “blood lipid” OR “plasma lipid” OR “blood fat” OR Lipemia OR “Lipidemia OR Hyperlipemia OR Hyperlipidemia OR Hypolipemia OR Hypolipidemia OR Cholesterol OR Triglyceride OR Triacylglycerol OR lipoprotein OR lipoproteinemia OR HDL OR “high-density lipoprotein” OR “LDL” OR “low-density lipoprotein”) AND (Dialysis OR Hemodialysis OR Hemodialysis OR hemodiafiltration OR hemofiltration OR “renal replacement therapy” OR “kidney failure” OR “renal failure” OR “dialysis solutions” OR “chronic kidney disease” OR “CKD” OR “chronic renal disease” OR “CRD” OR “end-stage renal disease” OR “ESRD” OR “end-stage kidney disease” OR “ESKD”). In addition to electronic database searches, manual searches were performed using Google Scholar, and the reference lists of all relevant primary and secondary sources were screened to identify additional eligible studies and gray literature.

Two independent reviewers (S.R. and N.SH.) screened and assessed all retrieved studies for eligibility based on predefined inclusion and exclusion criteria. Any disagreements were resolved through consultation with a third reviewer (M.K.), who provided a final assessment to ensure the accuracy and completeness of the selected studies.

### 2.2 Inclusion and exclusion criteria

Two reviewers (M.K. & S.R.) assessed the retrieved articles separately to determine their eligibility. The framework for eligibility criteria in this systematic review study was formulated according to the Population, Intervention, Comparison, and Outcomes (PICO) criteria (22) (Table 1). Inclusion criteria for this systematic review and meta-analysis were as follows: (1) randomized controlled trials (RCTs) that investigated the effects of LC supplementation on lipid profiles in adult patients undergoing hemodialysis; (2) studies that reported specific outcomes related to lipid profiles, including total cholesterol (TC), triglycerides (TG), high-density lipoprotein (HDL), low-density lipoprotein (LDL), and very low-density lipoprotein (VLDL); (3) studies published in peer-reviewed journals; and (4) studies published in English from inception to June 2024.

**TABLE 1:** The population, intervention, comparison, outcome, study design (PICO) criteria.

Domain	Criteria selection
Participants	Adult patients undergoing hemodialysis patients
Intervention group	L-carnitine
Comparison group	Placebo, control
Outcomes	Lipid profile (LDL, HDL, TG, TC, VLDL)

Exclusion criteria included: (1) studies involving populations other than adult patients undergoing hemodialysis; (2) non-randomized trials, observational studies, case reports, or reviews; (3) studies that did not include a control group; (4) trials that did not report relevant lipid profile outcomes; and (5) studies that assessed LC in combination with other interventions without a clear distinction of effects. These criteria ensured a focused analysis of the available evidence regarding the efficacy of LC supplementation on lipid profiles in the specified population.

### 2.3 Data extraction

Data extraction was performed independently by two reviewers using a standardized form to ensure consistency and accuracy. Key information extracted from each included study encompassed study characteristics (first author, year of publication, study design, sample size, and participant demographics), intervention details (dosage, route of administration, treatment duration, and control conditions), and primary outcomes related to lipid profiles, specifically TC, TG, HDL, LDL, and VLDL. Secondary outcomes, such as body mass index (BMI) and blood pressure (BP), were also documented where available. Data on study quality indicators, including risk of bias (e.g., random sequence generation, allocation concealment, blinding), were also collected to facilitate further analysis. Discrepancies between reviewers (S.P. & N.SH.) were resolved through discussion, with a third reviewer (M.K.) consulted if necessary, and the extracted data were compiled for analysis to evaluate the effects of LC supplementation on the lipid profiles of hemodialysis patients.

### 2.4 Quality assessment

The quality of RCTs was assessed for bias using the Cochrane risk of bias (RoB-1). Each study was rated as having low, some concerns, or high risk in various domains, including random sequence generation, allocation concealment, selective reporting, blinding of participants and personnel, blinding of outcome assessment, incomplete outcome data, and other biases (23, 24). Any disagreements were resolved through consensus.

### 2.5 Statistical analysis

The mean change and SD between baseline and last follow-up for TG, TC, HDL, LDL, VLDL, BMI, systolic BP, and diastolic BP in the intervention and control arms were extracted. The standardized mean difference (SMD) and 95% confidence interval (CI) used to compare the effect size (25). Some studies reported median and interquartile range (IQR) or median and range of variables. Luo et al.’s (26) and Wan et al.’s (27) methods were utilized to convert that report into mean and SD. Once the SD of the mean change was not reported directly, the following formula was used: SD change = square root [(SD baseline^2^ + SD final^2^) – (2 × 0.5 × SD baseline × SD final)] (28). Our meta-analyses employed a random-effects model using restricted maximum likelihood estimation. The between-study heterogeneity was assessed using Cochrane’s Q statistic and Hedges’ g I^2^ estimation (29). In the current analysis, we classified I^2^ values less than 25% as low heterogeneity, values between 25 and 50% as moderate heterogeneity, and values exceeding 50% as high heterogeneity. We also performed subgroup analysis based on the route of administration (RoA; IV vs Oral). Visual and statistical assessments using funnel plots and Begg’s and Egger’s tests were performed regarding the risk of publication bias (30, 31). Meta-regression analysis was conducted for variables reported in more than ten articles, including the year of publication, dosage of L-carnitine, and duration of treatment. All analyses were performed using R Statistical Software [v4.1.2; R Core Team (32)].

## 3 Results

### 3.1 Study selection

**FIGURE 1:**
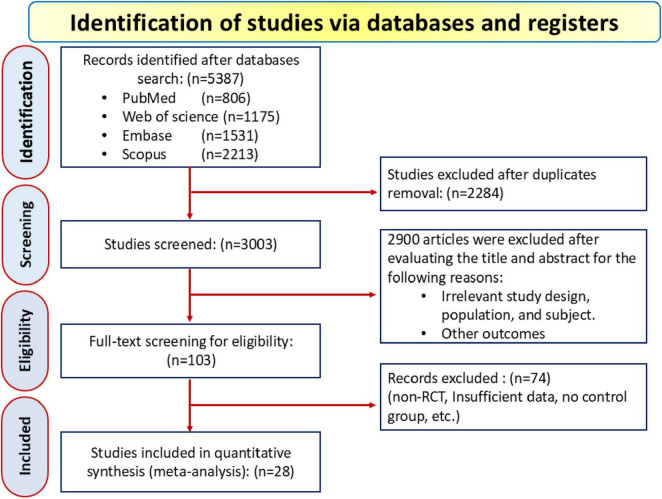
Flow chart of selection of studies for inclusion in meta-analysis.

A total of 5,387 records were initially identified—806 from PubMed, 1,531 from Embase, 1,175 from Web of Science, and 2,213 from Scopus—and after removing 2,284 duplicates, 3,003 studies were screened based on title and abstract. During this process, 2,900 records were excluded for reasons including irrelevant study designs (e.g., observational studies, case reports), non-hemodialysis patients, lack of focus on LC supplementation, or absence of relevant lipid profile outcomes. This left 103 eligible articles for full-text review, with 28 RCT ultimately included in the final analysis, as depicted in the PRISMA flow diagram in Figure 1.

### 3.2 Study characteristics

Table 2 details the included studies. All the RCT included in the study were published between 1980 and 2024. A total of 1,340 patients under hemodialysis treatment were studied, including 671 cases in the intervention arm and 669 subjects in the control arm. The most common etiology of hemodialysis was ESRD (17 studies), followed by CRF (8 studies) and Uremic conditions (1 study). The etiology was not reported in 2 articles. The duration of the included articles ranged from 5 to 48 weeks, and the sample size ranged from 10 to 148 cases.

**TABLE 2:** Basic characteristics of included studies.

References	Study design	Population patients	Etiology	Sample size (intervention/ control)	Age (year)	Male/female (%)	Intervention/ dose	RoA	Control	Duration	Lipid profile outcome
Guarnieri et al. (54)	RCT, single-blind	Hemodialysis	CRF	16 (8/8)	24–66	NR	L-Carnitine 0.5–1 gr/day	IV	Placebo	14 weeks	↓ TG, − TC
Weschler et al. (55)	RCT, double-blind	Hemodialysis	Uremic	10 (6/4)	36–66	80/20	L-Carnitine 3 gr/day	Oral	Placebo	5 weeks	↑ TG, − TC, − LDL, − HDL, − VLDL
Nilsson-Ehle et al. (56)	RCT, double-blind	Hemodialysis	NR	28 (14/14)	24–65	NR	L-Carnitine 2 gr/day	IV	Placebo	6 weeks	− TG, − TC, − HDL, − LDL
Yderstræde et al. (57)	RCT, double-blind	Hemodialysis	ESRD	21 (11/10)	20–72	72.9/27.1	L-Carnitine 100 mumol/l	IV	Placebo	24 weeks	↓ TG, − TC, − HDL, − LDL, − Apolipoprotein
Golper et al. (58)	RCT, double-blind	Hemodialysis	ESRD	82 (38/44)	LC: 47.5 CG: 48	63.2/36.8	L-Carnitine 20 mg/kg	IV	Placebo	24 weeks	− TG, − TC, − HDL, − LDL, − VLDL, − Apolipoprotein
Labonia (59)	RCT, double-blind	Hemodialysis	ESRD	24 (13/11)	LC: 41.8 CG: 62.5	46.2/53.8	L-Carnitine 1 gr/day	IV	Placebo	24 weeks	TG, TC, HDL
Vaux et al. (60)	RCT, double-blind	Hemodialysis	ESRD	26 (13/13)	LC: 58.8 CG: 63.8	76.9/23.1	L-Carnitine 20 mg/kg	IV	Placebo	16 weeks	− TG, − TC
Mitwalli et al. (61)	RCT, single-blind	Hemodialysis	ESRD	31 (18/13)	LC: 54 CG: 42	38.9/61.1	L-Carnitine 15 mg/kgI	IV	Placebo	24 weeks	↓ TG, ↓TC,
Steiber et al. (62)	RCT, double-blind	Hemodialysis	ESRD	34 (15/19)	LC: 67.6 CG: 69.4	46.7/53.3	L-Carnitine 20/kg	IV	Placebo	24 weeks	− TG, HDL
Rathod et al. (63)	RCT, single-blind	Hemodialysis	ESRD	20 (10/10)	LC: 40.3 CG: 47.3	M: 100%	L-Carnitine 20 mg/kg	IV	Placebo	8 weeks	− TG, − TC, − HDL, − LDL
Duranay et al. (64)	RCT, open-label	Hemodialysis	ESRD	42 (21/21)	LC: 44 CG: 43.4	61.9/38.1	L-Carnitine 20 mg/kg	IV	Control	24 weeks	TG, TC, LDL
Sakurabayashi et al. (65)	RCT, open-label	Hemodialysis	ESRD	20 (10/10)	LC: 45.7 CG: 46	90/10	L-Carnitine 10 mg/kg	Oral	Control	48 weeks	− TG, − TC, − HDL
Shakeri et al. (66)	RCT, unblinded	Hemodialysis	CRF	36 (18/18)	LC: 54.5 CG: 57	66.7/33.3	L-Carnitine 1 gr/day	Oral	Control	12 weeks	↓ TC
Shojaei et al. (67)	RCT, double-blind	Hemodialysis	NR	25 (12/13)	LC: 55.3 CG: 51.6	50/50	L-Carnitine 1 gr/day	IV	Placebo	12 weeks	− TG, − TC, − HDL, − LDL
Suchitra et al. (68)	RCT, single-blind	Hemodialysis	ESRD	35 (20/15)	LC: 50.2 CG: 53.4	65/35	L-Carnitine 1 gr/day	IV	Control	24 weeks	− TG, − TC, − HDL, − LDL, − VLDL
Mercadal et al. (33)	RCT, double-blind	Hemodialysis	CRF	92 (46/46)	LC: 61 CG: 61	NR	L-Carnitine 1 gr/day	IV	Placebo	48 weeks	− TG, − TC, − HDL, − LDL
Mortazavi et al. (69)	RCT, double-blind	Hemodialysis	ESRD	36 (17/19)	>21	51.9/48.1	L-Carnitine 0.75 gr/day	Oral	Placebo	24 weeks	− TG, − TC, − HDL, − LDL
Naini et al. (70)	RCT	Hemodialysis	ESRD	60 (30/30)	21–78	63.3/36.7	L-Carnitine 0.75 gr/day	Oral	Control	8 weeks	↓ TG, ↓ TC, − HDL, ↓ LDL
Emami Naini et al. (71)	RCT, double-blind	Hemodialysis	ESRD	51 (24/27)	LC: 53.9 CG: 51.85	50/50	L-Carnitine 1 gr/day	Oral	Placebo	16 weeks	↓ TG, − TC, ↑ HDL, − LDL
Fukami et al. (72)	RCT, open-label	Hemodialysis	CRF	70 (32/38)	LC: 68 CG: 67	68.8/31.3	L-Carnitine 0.9 gr/day	Oral	Control	24 weeks	↑ TG, − HDL, ↑ LDL
Higuchi et al. (73)	RCT, open-label	Hemodialysis	CRF	131 (67/64)	LC: 67 CG: 68	71.5/28.5	L-Carnitine 20 mg/kg	Oral	Control	12 weeks	− TG, − TC, − LDL
Eshghnia et al. (74)	RCT, double-blind	Hemodialysis	ESRD	34 (17/17)	LC: 45.1 CG: 43.12	NR	L-Carnitine 1 gr/day	Oral	Placebo	16 weeks	↓ TG, − TC, − HDL, − LDL, ↓ VLDL
Kudoh et al. (75)	RCT, double-blind	Hemodialysis	CRF	15 (9/6)	LC: 66.2 CG: 70.5	33.3/66.7	L-Carnitine 0.9 gr/day	Oral	Placebo	12 weeks	− TG, − TC, − HDL, − LDL
Higuchi et al. (76)	RCT, open-label	Hemodialysis	ESRD	148 (75/73)	LC: 66 CG: 67	80/20	L-Carnitine 20 mg/kg	Oral	Control	48 weeks	− TG, − TC, − LDL
Maruyama et al. (77)	RCT, open-label	Hemodialysis	CRF	60 (30/30)	LC: 70 CG: 69	70/30	L-Carnitine 1 gr/day	IV	Control	48 weeks	− TG, − TC, − LDL
Mohammadi-Baneh et al. (78)	RCT, double-blind	Hemodialysis	RF	71 (35/36)	LC: <60: 14/ ≥60: 21 CG: <60: 19/ ≥60: 17	57.1/42.9	L-Carnitine 1 gr/day	Oral	Placebo	12 weeks	− TG, − TC
Sugiyama et al. (79)	RCT, single-blind	Hemodialysis	ESRD	35 (18/17)	L: 64.5 C: 69.5	66.66/33.33	L-Carnitine 1 gr/day	IV	Placebo	24 weeks	↑ TC, ↑ LDL
Shayanpour et al. (80)	RCT, double-blind	Hemodialysis	ESRD	87 (44/43)	LC: 50.27 CG: 49.04	52.27/47.73	L-Carnitine 0.5 gr/day	Oral	Placebo	12 weeks	− LDL

RCT, randomized controlled clinical trial; RoA, rout of administration; ESRD, end-stage renal disease; CRF, chronic renal failure; RF: renal failure; TG, triglyceride; TC, total cholesterol; LDL, low-density lipoprotein; HDL, high-density lipoprotein; VLDL, very low-density lipoprotein; LC, L-carnitine group; CG, control group; IV, intravenous; NR, not reported.

### 3.3 Risk of bias in studies

**FIGURE 2:**
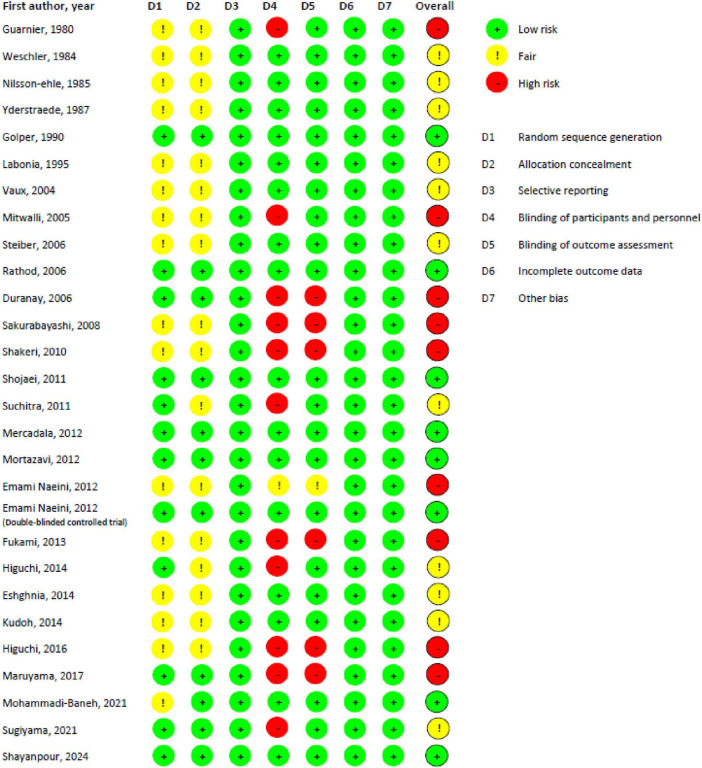
Risk of bias according to ROB-1 tool for randomized trials.

**FIGURE 3:**
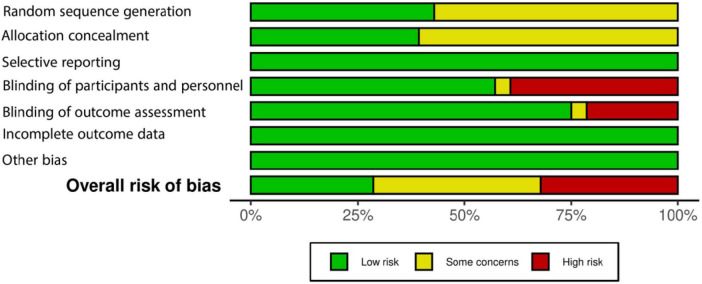
The risk of bias graph for randomized controlled trials regarding ROB-1.

Figures 2, 3 present the risk of bias assessment results. Eleven studies contained some concerns about the risk of bias. Nine articles were classified as high risk, and eight studies as low risk of biased papers.

### 3.4 Effect of L-carnitine on triglycerides (TG)

**FIGURE 4:**
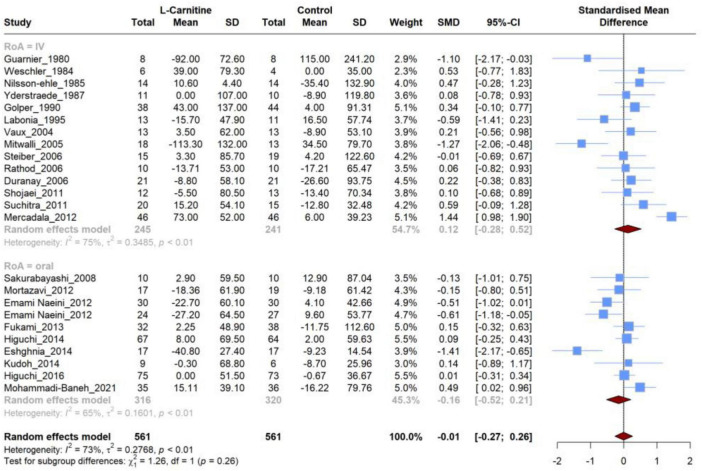
Forest plot for meta-analysis of triglycerides (TG) mean change in L-carnitine groups versus control groups.

Twenty-four articles were included that compared the efficacy of LC on TG levels versus placebo. Meta-analysis of these studies demonstrated (Figure 4) that there was no significant difference in the mean changes (follow-up from baseline) between groups (SMD, −0.006; 95% CI, −0.272 to 0.259; *P* = 0.95), and this analysis had a high heterogeneity (I^2^ = 73.5%). Additionally, there was no significant (*P* = 0.26) between-group difference in subgroup analysis based on the RoA (IV *vs.* oral). Furthermore, none of these two subgroups showed significant differences (Table 3) in the mean changes (IV: SMD, 0.12; 95% CI, −0.28 to 0.52; Oral: SMD, −0.01; 95% CI, −0.27 to 0.26), while heterogeneity remained high for both subgroups (75% and 65%, respectively). Further subgroup analyses based on the dosage and duration of treatment also demonstrated no significant SMD in any subgroups (Supplementary Figure 1).

**TABLE 3:** Summary effects of L-carnitine and subgroup analysis on the route of L-carnitine supplementation on outcomes of interest among hemodialysis patients.

Variable	Number of studies	SMD	95% CI (lower limit)	95% CI (upper limit)	*P*-value^1^	Heterogeneity (I^2^)	*P*-value^2^	Risk of publication bias (*p*-value^3^)
TG	24	−0.0065	−0.2721	0.2590	0.95	73.5%	<0.000	0.20
TC	24	−0.0866	−0.2530	−0.0797	0.29	32.5%	0.064	0.89
HDL	17	0.0601	−0.0575	0.1777	0.294	0.0%	0.949	0.97
LDL	18	−0.0649	−0.2727	0.1428	0.518	53.7%	0.003	0.39
VLDL	4	−0.1255	−1.2716	1.0206	0.750	76.4%	0.005	NA
BMI	5	−0.0258	−0.1396	0.0881	0.564	0.0%	0.986	NA
Systolic BP	6	0.0552	−0.1106	0.2209	0.431	0.0%	0.844	NA
Diastolic BP	5	−0.0288	0.1569	0.0994	0.567	0.0%	0.943	NA

TG, triglyceride; TC, total cholesterol; HDL, high-density lipoprotein; LDL, low-density lipoprotein; BP, blood pressure.

^1^*p*-value of variables SMD.

^2^*p*-value of heterogeneity based on Cochran’s Q.

^3^*p*-value of risk of publication bias assessment by Egger’s method. *SMD, standardized mean difference; CI, confidence interval.

A meta-regression analysis was performed using several continuous variables, such as publication year, dosage, and treatment duration. Table 4 demonstrates the detailed result of this analysis. None of the variables were significantly associated with pooled effect size.

**TABLE 4:** Findings of meta-regression analysis of TG, TC, HDL, and LDL with year of publication, dosage of L-carnitine, and duration of treatment.

Variables	Meta-regression	Heterogeneity (I^2^)	Residual heterogeneity (I^2^)	Test of moderator (*P*-value)
TG	Year of publication	0.0%	75.77%	0.82
	Dosage	3.41%	75.64%	0.17
	Duration	15.04%	71.44%	0.14
TC	Year of publication	0.0%	34.96%	0.66
	Dosage	5.86%	32.7%	0.34
	Duration	41.01%	20.95%	0.19
HDL	Year of publication	0.0%	0.0%	0.88
	Dosage	0.0%	0.0%	0.71
	Duration	0.0%	0.0%	0.19
LDL	Year of publication	0.0%	58.13%	0.73
	Dosage	0.0%	59.99%	0.86
	Duration	30.64	46.24%	0.08

TG, triglyceride; TC, total cholesterol; HDL, high-density lipoprotein; LDL, low-density lipoprotein.

### 3.5 Effect of L-carnitine on total cholesterol (TC)

**FIGURE 5:**
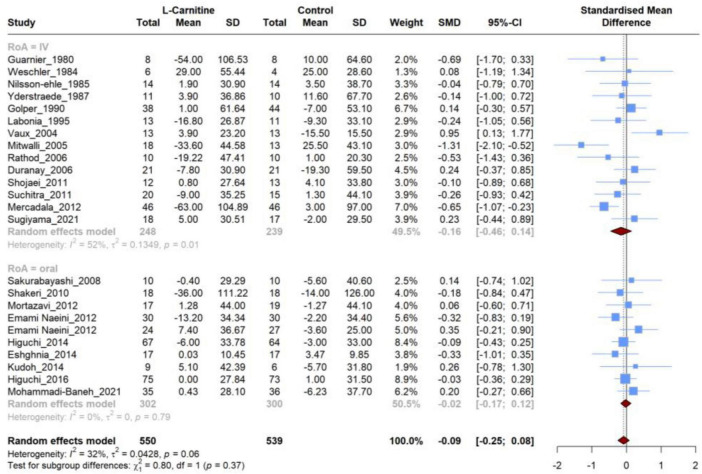
Forest plot for meta-analysis of total cholesterol (TC) mean change in L-carnitine groups versus control groups.

A total of 24 RCT were incorporated in the meta-analysis, all of which compared the effect of LC on TC levels to placebo. The meta-analysis of these studies resulted in no significant (SMD, −0.086; 95% CI, −0.253 to −0.079; *P* = 0.29) difference in the mean changes between groups (Figure 5). In addition, the heterogeneity of studies was moderate (I^2^ = 32.5%).

The subgroup analysis based on the RoA did not reveal significant differences between the groups (*P* = 0.37). Furthermore, neither of these two subgroups exhibited significant differences regarding the mean changes (IV: SMD, −0.16; 95% CI, −0.46 to 0.14; Oral: SMD, −0.02; 95% CI, −0.17 to 0.12). However, heterogeneity was high for the IV subgroup (I^2^ = 52%), and the oral subgroup contained homogeneous studies (I^2^ = 0%) (Table 3). Similar to the RoA, additional investigation based on subgroup analysis of dosage and duration of treatment revealed no significant SMD in subgroups (Supplementary Figure 2).

Results from meta-regression analysis showed that the duration of treatment accounted for 41.01% of heterogeneity among studies. In contrast, the moderator test for the duration was non-significant (*P* = 0.19). These findings showed that although the duration of treatment could explain some of the heterogeneity, it did not help predict the SMD between arms of studies. Furthermore, the year of publication and dosage were found not to be a source of heterogeneity (Table 4).

### 3.6 Effect of L-carnitine on HDL, LDL, VLDL

**FIGURE 6:**
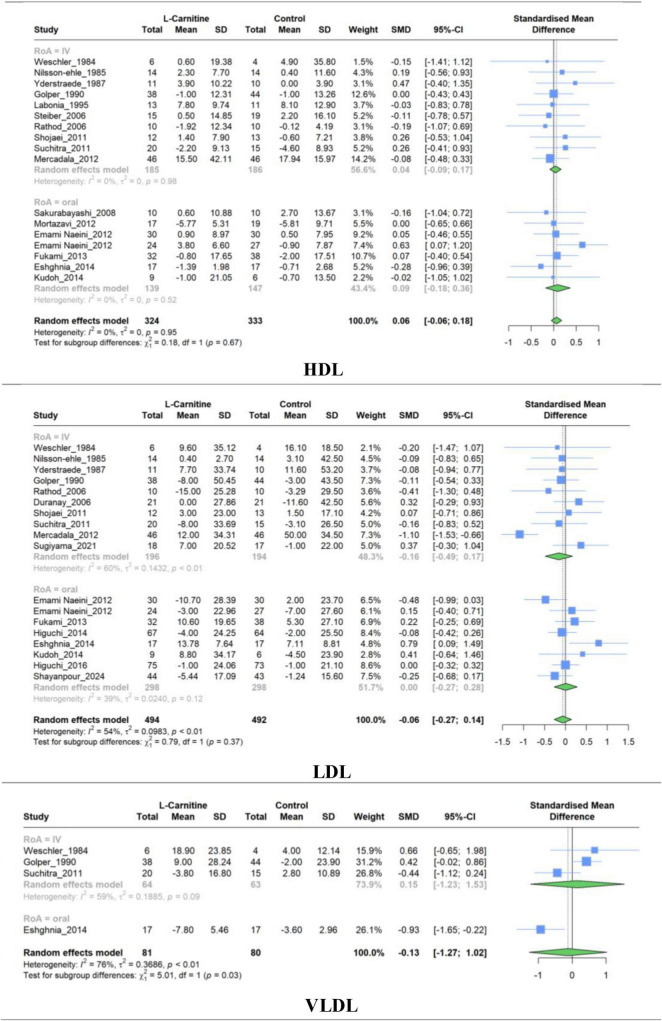
Forest plot for meta-analysis of HDL, LDL, and VLDV mean change in L-carnitine groups versus control groups.

A meta-analysis of studies comparing LC and placebo effectiveness in modifying HDL, LDL, and VLDL levels was conducted separately, and it comprised 17,18 and 4 studies, respectively. Similar to the previous lipid profile variables, no significant difference was observed in the mean changes between LC and placebo for HDL levels (SMD, 0.060; 95% CI, −0.057 to 0.177; *P* = 0.29), LDL levels (SMD, −0.064; 95% CI, −0.272 to 0.142; *P* = 0.51), and VLDL (SMD, −0.125; 95% CI, −1.271 to 1.020; *P* = 0.75) (Figure 6). Regarding the HDL levels, there was no between-study heterogeneity (I^2^ = 0.0%). However, analysis of LDL and VLDL levels revealed high heterogeneity (I^2^ = 53.7% and 76.4%, respectively).

Similar to the overall pooled effect for HDL studies, the subgroup synthesized results based on the RoA were non-significant (IV: SMD, 0.03; 95% CI, −0.09 to 0.15; Oral: SMD, 0.08; 95% CI, −0.18 to 0.36). Furthermore, no significant (*P* = 0.67) between-group differences were observed (Table 3).

Subgroup meta-analysis evaluating the efficacy of LC on LDL resulted in non-significant pooled effects of 10 studies with IV administration (SMD, −0.16; 95% CI, −0.48 to 0.16) and eight studies with oral administration (SMD, 0.00; 95% CI, −0.27 to 0.28). Supplementary Figures 3–5 represent subgroup analyses of dosage and treatment duration for HDL, LDL, and VLDL, respectively. The findings of subgroup analyses were similar to the overall results for each variable and revealed no significant SMD.

Meta-regression with the same variables (year of publication, dosage, and duration) was performed for HDL and LDL analysis. The results of the HDL analysis were non-significant, and the pooled estimate was not associated significantly with other variables. On the other hand, the variance between studies was partially attributed to the treatment duration, accounting for 30.64% of the heterogeneity. Nevertheless, the duration could not be a significant moderator of SMD between groups (*P* = 0.08). The outcomes of other variables’ meta-regression were non-significant (Table 4).

### 3.7 Effect of L-carnitine on BMI and BP

**FIGURE 7:**
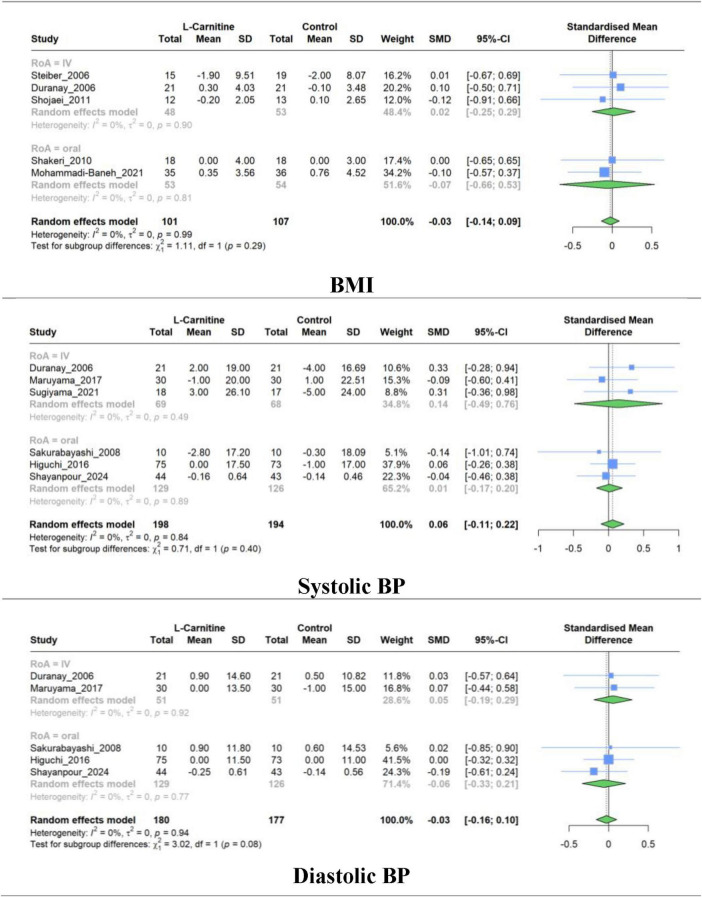
Forest plot for meta-analysis of BMI, systolic BP, and diastolic BP mean change in L-carnitine groups versus control groups.

Meta-analysis of studies evaluating the effect of LC on BMI (5 RCT), systolic BP (6 RCT), and diastolic BP (5 RCT) was performed separately. The combined results show that there was no significant difference in the mean change between intervention and control groups for BMI (SMD, −0.025; 95% CI, −0.139 to 0.088; *P* = 0.56), systolic BP (SMD, 0.055; 95% CI, −0.110 to 0.220; *P* = 0.43), and diastolic BP (SMD, −0.028; 95% CI, 0.156 to 0.099; *P* = 0.56). The studies exhibited very low heterogeneity in all groups (I^2^ = 0.0%) (Figure 7). The results of subgroup analysis based on the RoA, treatment duration, and dosage were non-significant (Supplementary Figures 6–8).

### 3.8 Sensitivity analysis

**FIGURE 8:**
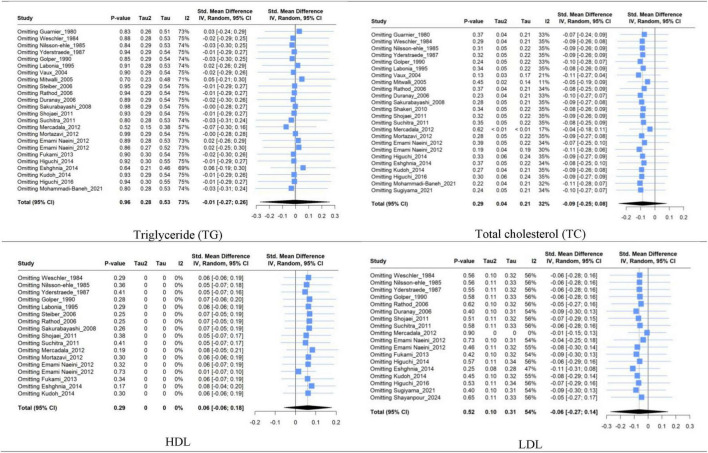
Forest plot for sensitivity analysis based on leave-one-out method of triglyceride (TG), total cholesterol (TC), high-density lipoprotein (HDL), and low-density lipoprotein (LDL) between mean change in L-carnitine groups versus control groups.

Sensitivity analysis based on the leave-one-out method for TG, TC, and HDL studies showed that excluding articles did not significantly change heterogeneity or pooled results. However, the leave-one-out method’s finding in LDL revealed that the high between-study heterogeneity in the LDL group could be reduced significantly by removing Mercadal et al. (33) study. In contrast, removing any studies did not significantly change the LDL group pooled estimate (Figure 8).

The Supplementary materials include a Sensitivity analysis based on the leave-one-out method for systolic BP, diastolic BP, and BMI (Supplementary Figure 9).

### 3.9 Assessment of publication bias

**FIGURE 9:**
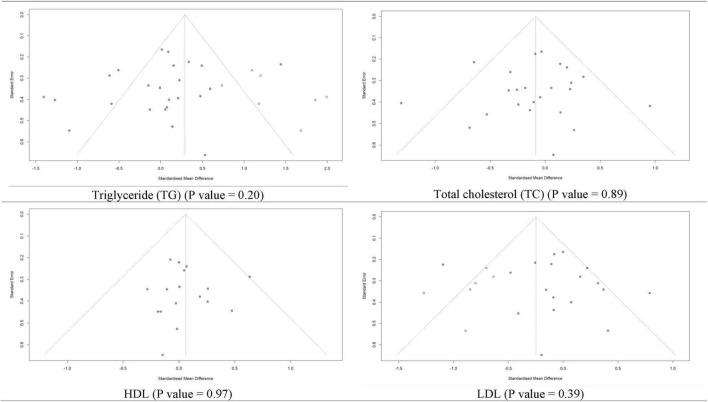
Funnel plot for risk of publication bias assessment based on trim and fill method of SMD of triglyceride, total cholesterol, high-density lipoprotein (HDL), and low-density lipoprotein (LDL) between L-carnitine groups versus control groups with *P*-value of Egger’s test.

Regarding the risk of publication bias, the funnel plot’s visual assessment did not show asymmetry or risk of bias for any of the analyses (Figure 9 and Supplementary Figures 10–13). Moreover, for a meta-analysis with more than ten included studies, statistical tests (Begg’s and Egger’s methods) for assessing the risk of publication bias were used and confirmed no source of publication bias (Table 3).

## 4 Discussion

In this meta-analysis, we showed that LC supplementation did not significantly change serum lipid levels, including TG, TC, HDL, LDL, or VLDL. While previous research has suggested that hemodialysis patients often have carnitine deficiency (14, 18–20), which may contribute to metabolic disturbances such as dyslipidemia, our findings indicate that LC supplementation alone is not effective in improving lipid metabolism or managing dyslipidemia in this specific population. These results highlight the need for further investigation into alternative therapeutic approaches for reducing cardiovascular risk in hemodialysis patients.

Carnitine has a crucial role in lipid metabolism, considering its involvement in the beta-oxidation of fatty acids and reducing the conversion of free fatty acids (FFA) to TG (34). Carnitine deficiency in hemodialysis individuals is linked to several factors, such as inadequate carnitine intake, reduced biosynthesis, and removal during hemodialysis (15, 35). Abnormal carnitine metabolism in these patients is correlated with various clinical conditions, particularly impaired cardiac function (36, 37). Cardiovascular diseases are a leading cause of death in chronic renal failure individuals on maintenance hemodialysis (38), which can be related to impairments in cardiac metabolism along with higher inflammation and oxidative stress status. This can lead to myocyte necrosis resulting from altered lipid metabolism and LC deficiency (39, 40). In this regard, numerous RCTs have explored the impact of LC supplementation on lipid profiles across various diseases, particularly in hemodialysis patients. However, the findings have been inconsistent with partly modest sample sizes (16).

Although previous studies have demonstrated that LC supplementation generally improves lipid profiles (41–43), but the effects of LC in Specific populations are controversial. The conflicting outcomes from various studies underscore the complexity of LC’s effects in different patient populations. This inconsistency may stem from variations in study design, dosage, and patient characteristics. For example, a similar study conducted by Huang et al. (16) analyzing twelve studies with a total of 391 hemodialysis patients which concluded that LC significantly lowers LDL but does not affect TC, HDL, or TG. In another study on patients with liver disease, Abbasnezhad et al. (34) Showed that LC reduces TC and TG but has no significant effect on HDL and LDL. Asbaghi et al. (44) and Vidal-Casariego et al. (45) showed that in type 2 diabetes patients, LC improves TC and LDL-C levels but has no significant effect on HDL-C and TG. Yang et al. (17) in a meta-analysis, reported that LC therapy did not improve oxidized LDL (SMD: 0.04, *P* = 0.87) or TC (-0.24, P = 0.33). Also, in a meta-analysis by Huang et al. (16), LC supplementation did not significantly reduce TC, HDL, VLDL, or serum TG. However, it significantly decreased LDL in hemodialysis patients (SMD: −0.29, *P* = 0.01). In another systematic review (46) of patients with CKD, LC was found to increase levels of TC and LDL significantly.

In our meta-analysis, subgroup analysis based on the RoA, dosage, and duration of LC supplementation revealed no significant effects on TC, TG, HDL, or VLDL levels in hemodialysis patients. However, a notable reduction in LDL was observed in patients receiving intravenous LC. Regarding the dosage of LC, Musazadeh et al. (41) and Askarpour et al. (42), which reported that higher LC doses, particularly above 2 g/day, improved lipid profiles by reducing TC, LDL, and TG while increasing HDL. The discrepancies suggest that factors such as dosage and RoA might play a critical role in LC’s lipid-modifying effects, warranting further investigation to better understand its impact in different patient populations and treatment regimens.

Our study found that LC supplementation had no significant effect on BMI in hemodialysis patients, and subgroup analysis based on the RoA also showed no notable impact. This result aligns with findings from several other studies across different patient populations and conditions. For instance, Abolfathi et al. (47) reported no significant changes in BMI or body weight in patients with non-alcoholic fatty liver disease (NAFLD) following LC supplementation, while Del Vecchio et al. (48) similarly found no effect of LC on body mass reduction. However, contrasting outcomes were observed in studies by Pooyandjoo et al. (49) and Talenezhad et al. (50), where LC supplementation led to significant BMI reductions, particularly in overweight or obese individuals. These mixed results suggest that the effect of LC on BMI may vary based on factors such as patient population, baseline body weight, and underlying health conditions, highlighting the need for further research to clarify LC’s role in weight management.

The effect of LC on BP has shown mixed results in various studies. In our study, LC supplementation had no significant impact on either systolic or diastolic BP in hemodialysis patients, and subgroup analysis based on the RoA also yielded insignificant results. However, a meta-analysis by Askarpour et al. (51) reported that LC supplementation significantly reduced diastolic BP in overweight and obese participants (-1.232 mmHg, *P* = 0.023) and in those receiving doses less than 2 g per day (-1.639 mmHg, *P* = 0.022). In contrast, Choi et al. (52) found a significant reduction in systolic BP with LC supplementation, indicating potential variability between studies. Additionally, Dong et al. (53) observed that oral LC significantly lowers both systolic and diastolic BP, suggesting a broader range of cardiovascular effects for LC. These inconsistencies point to the need for further research to better understand how factors like patient population, dosage, and underlying health conditions may influence LC’s impact on BP.

This study has several strengths and limitations. A key limitation is the moderate to high heterogeneity across the included studies, which indicates variability in study designs, patient populations, and intervention protocols. Such variability can impact the reliability of the pooled results. Additionally, discrepancies in findings from other LC supplementation meta-analyses may be attributed to differences in population characteristics, types of interventions, sample sizes, study quality, and measurement methods, complicating the assessment of LC’s effects across different patient groups. On the other hand, the study’s strengths include its comprehensive analysis of LC supplementation’s effects on lipid profiles, BMI, and blood pressure in hemodialysis patients, offering a detailed examination of this specific population. The low to moderate heterogeneity in most outcomes enhances the reliability of the findings, and subgroup analyses based on dosage, duration, and route of administration provide further insights. By incorporating more variables and recent studies, this meta-analysis delivers a broader understanding compared to previous reviews.

## 5 Conclusion

In conclusion, LC supplementation does not significantly improve the serum lipid profile, including TG, TC, HDL, LDL, and VLDL, in hemodialysis patients. It also has no notable impact on BMI, systolic, or diastolic blood pressure in this population. Despite LC’s known role in lipid metabolism, the variability in findings across studies suggests that its effects may differ depending on patient populations, study design, dosage, and other factors. To reduce variation and get a clearer picture of the effects of LC supplementation, especially on managing cardiovascular risk, future research should focus on larger, well-designed RCTs with more specific patient groups.

## Data Availability

The original contributions presented in this study are included in this article/Supplementary material, further inquiries can be directed to the corresponding authors.
