# Supplementary material for: Effects of L-carnitine supplementation on lipid profile in adult patients under hemodialysis: a systematic review and meta-analysis of RCTs
Source: Front Med (Lausanne). 2024 Dec 2;11:1454921. doi: 10.3389/fmed.2024.1454921 (PMC11646722; doi:10.3389/fmed.2024.1454921)
Supplement: Supplementary file 1 [file Data_Sheet_1.docx]

**Suplementary Materials**

| 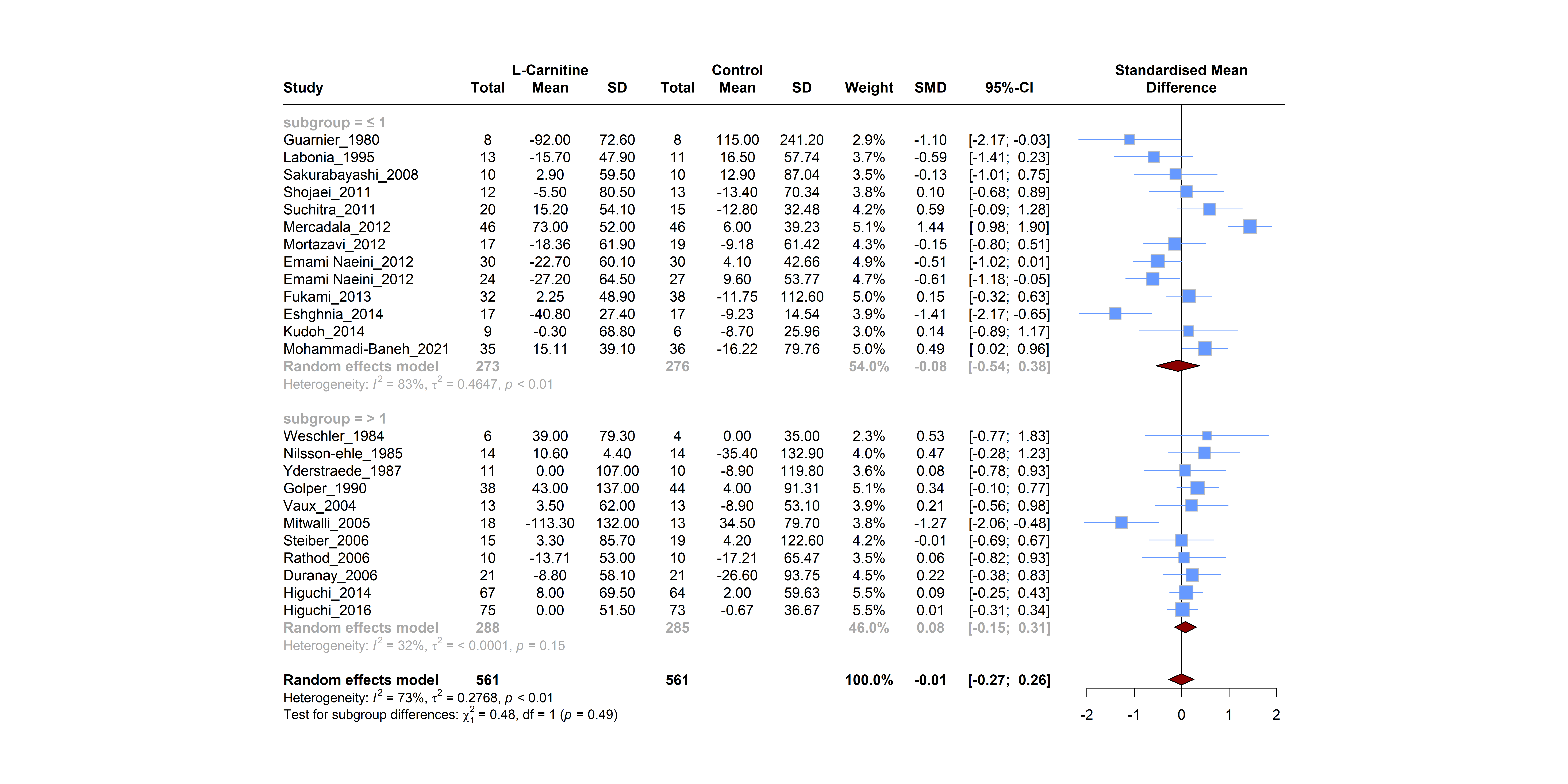  A: Dosage |
| --- |
| 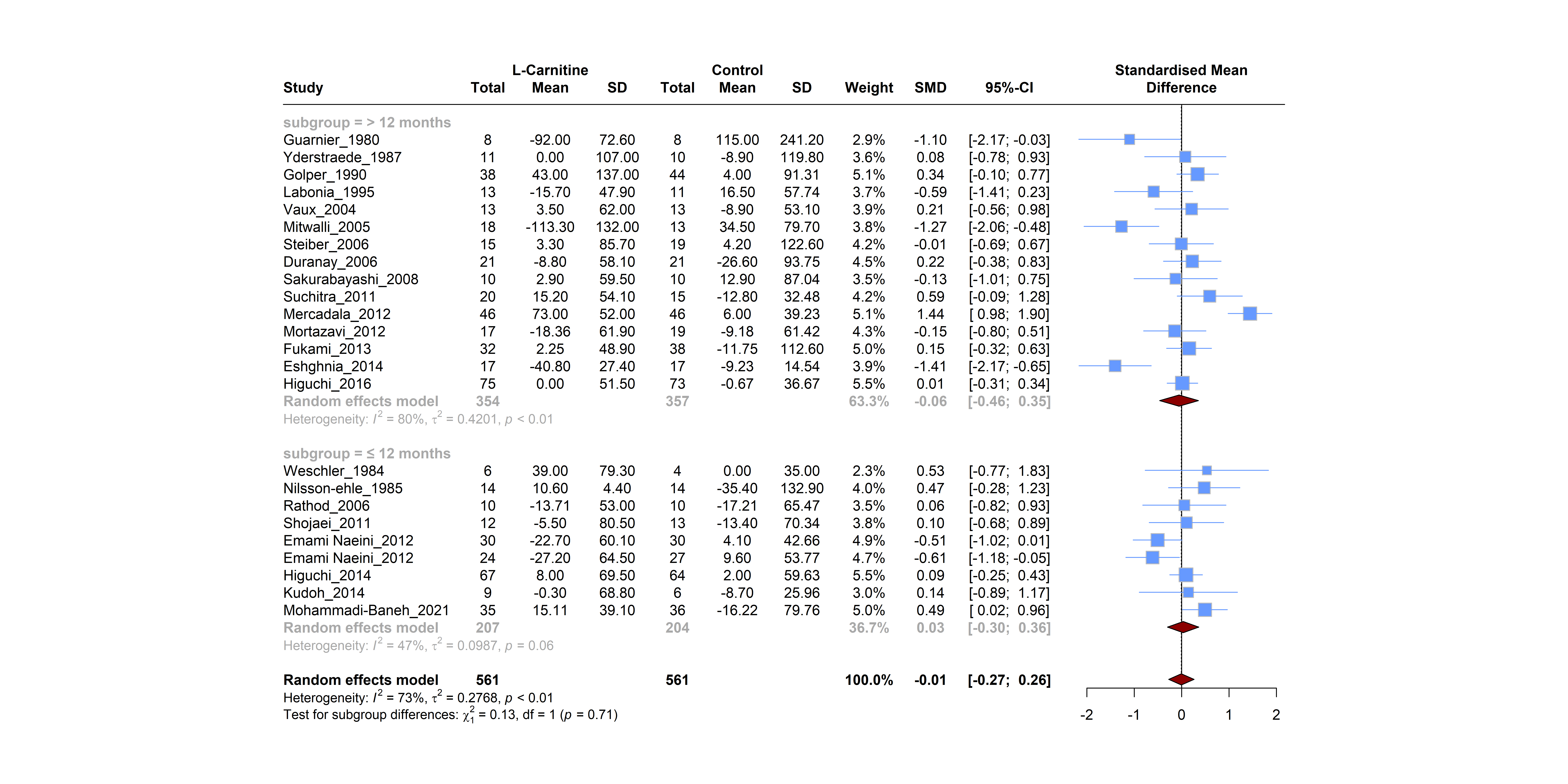  B: Duration |

**Figure S1.** Subgroup analysis based on the dosage (A) and treatment duration (B) for triglycerides (TG)

| 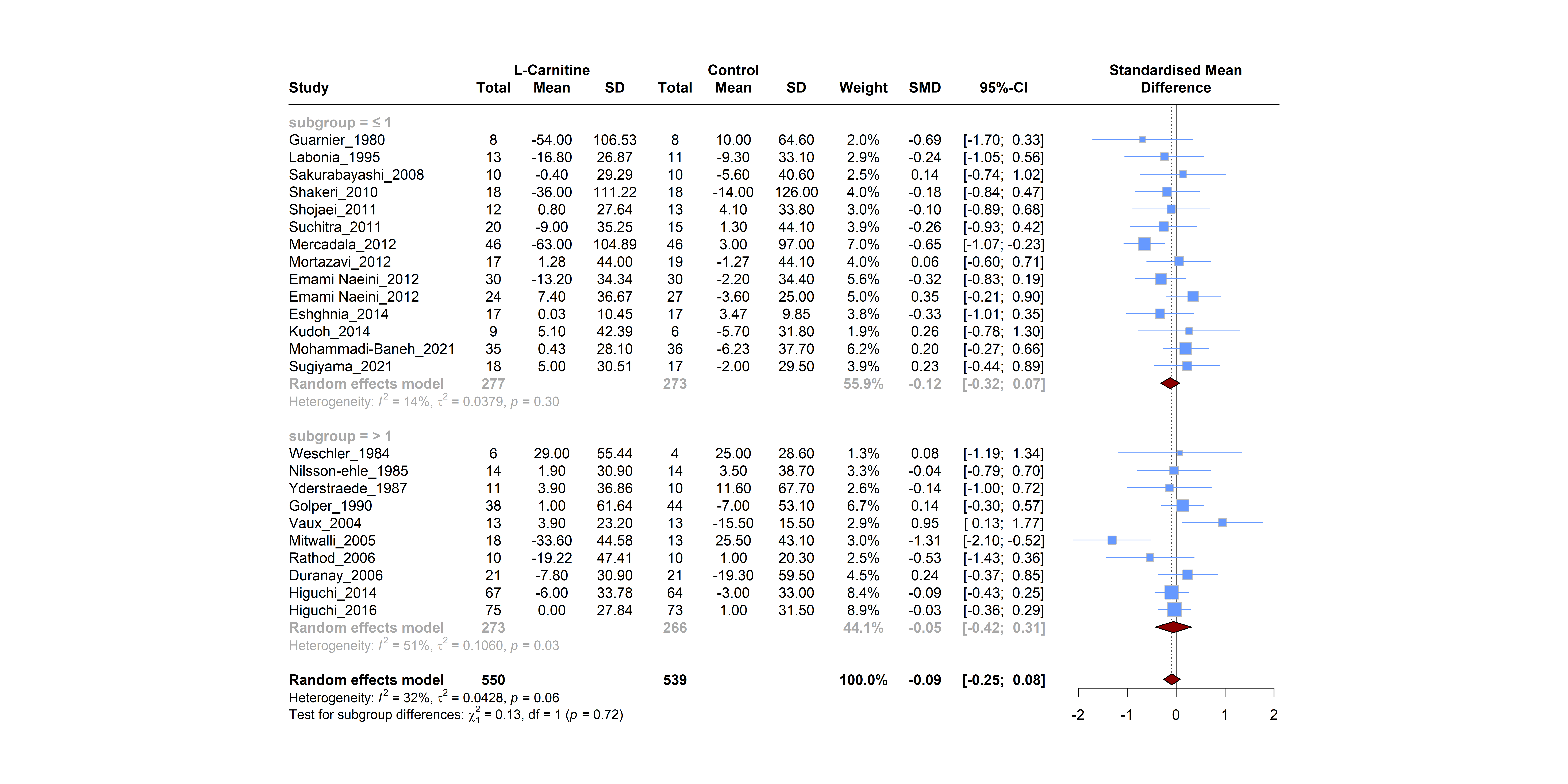  A: Dosage |
| --- |
| 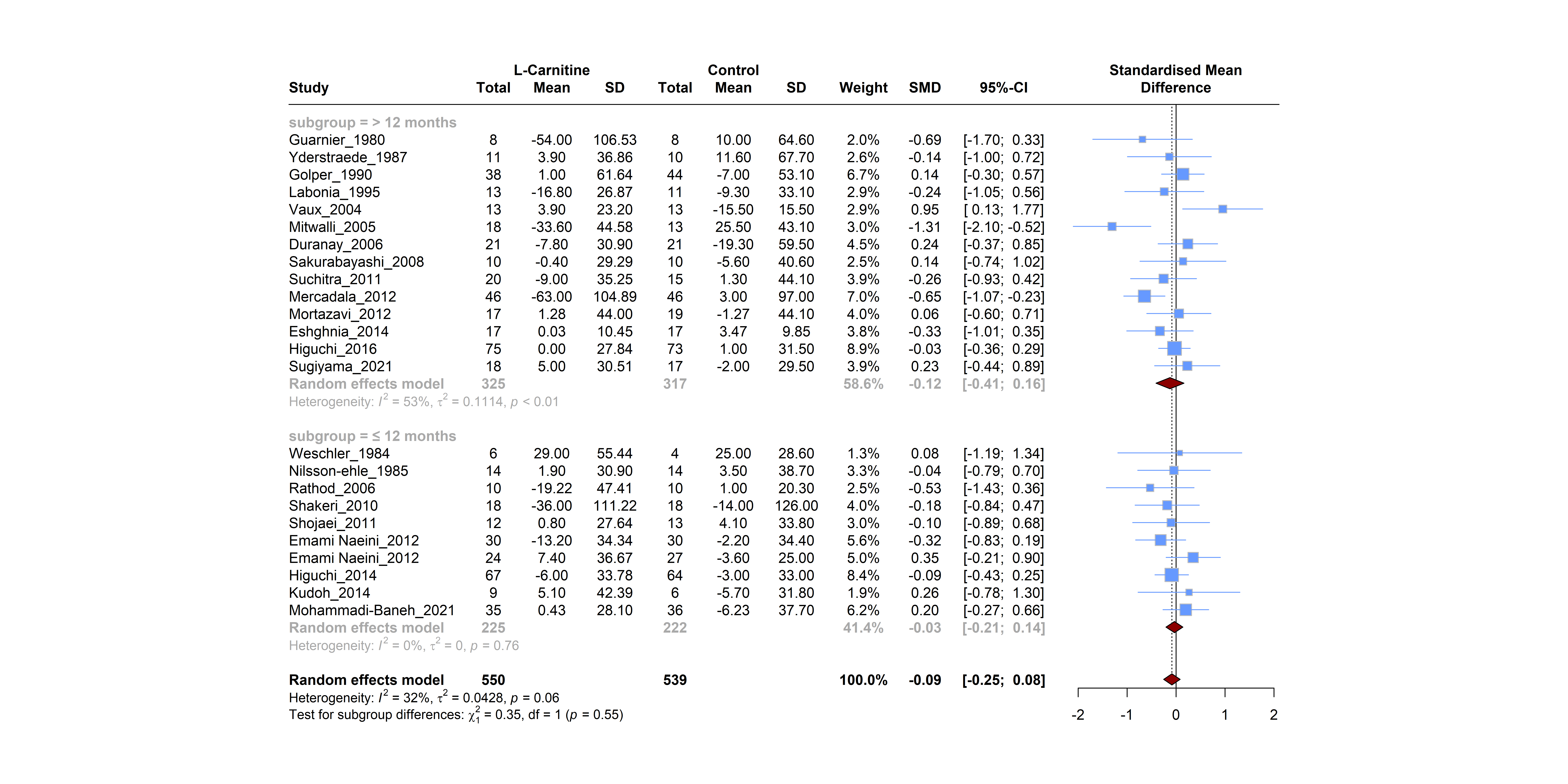B: Duration |
| 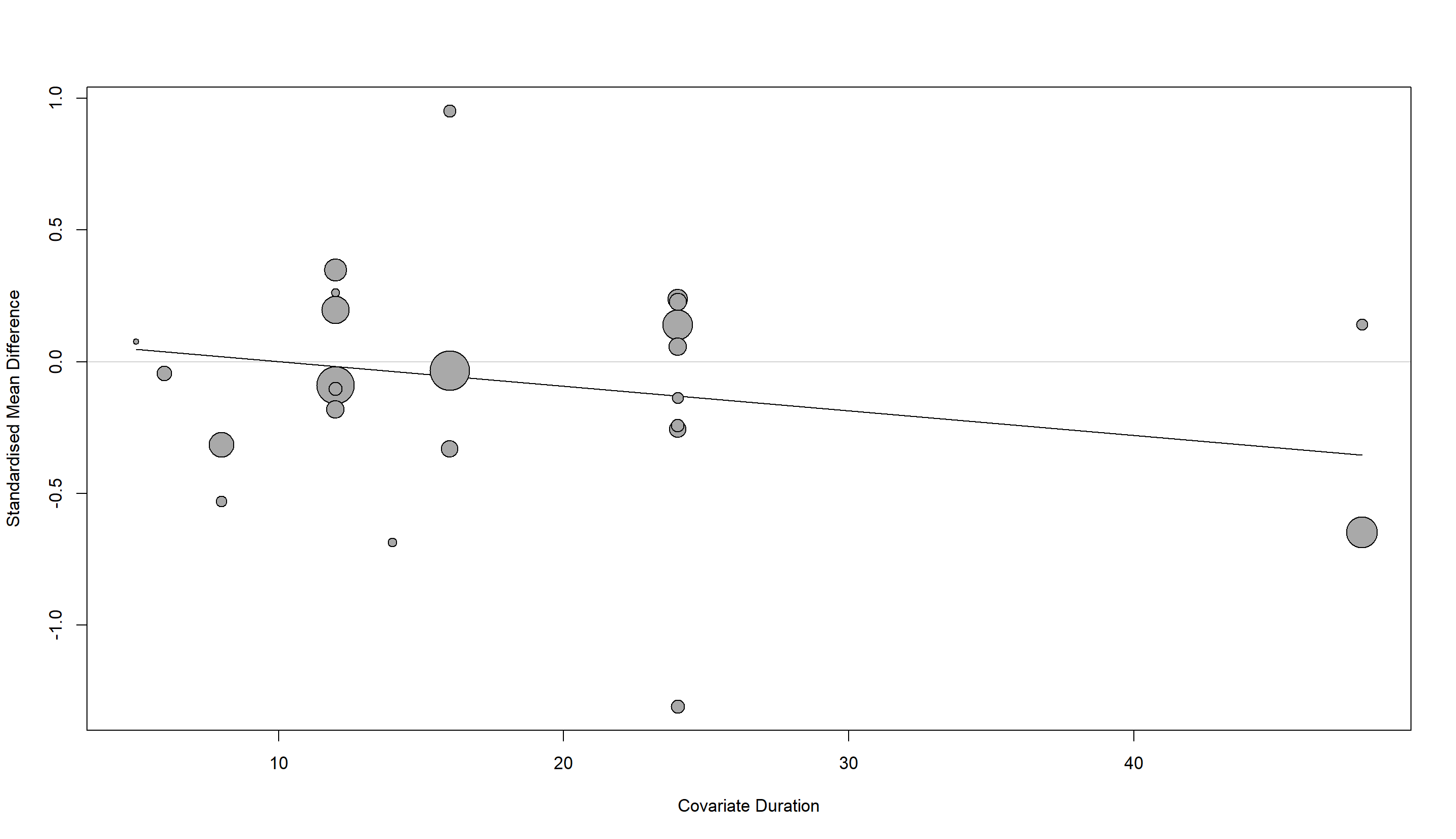  C: TC regression Covariable duration bubble |

**Figure S2.** Subgroup analysis based on the dosage (A) and treatment duration (B, C) for total cholesterol (TC)

| 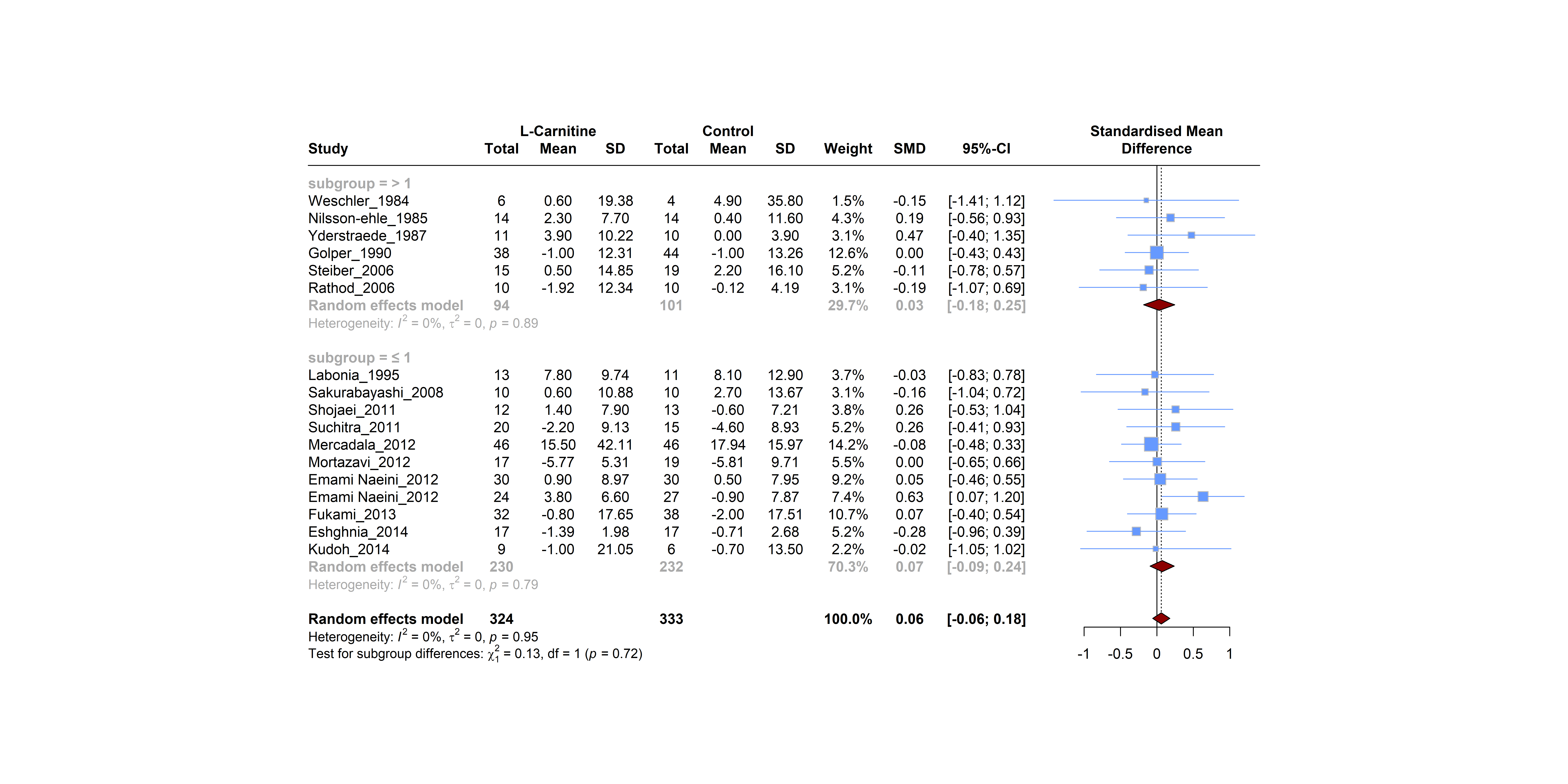  A: Dosage |
| --- |
| 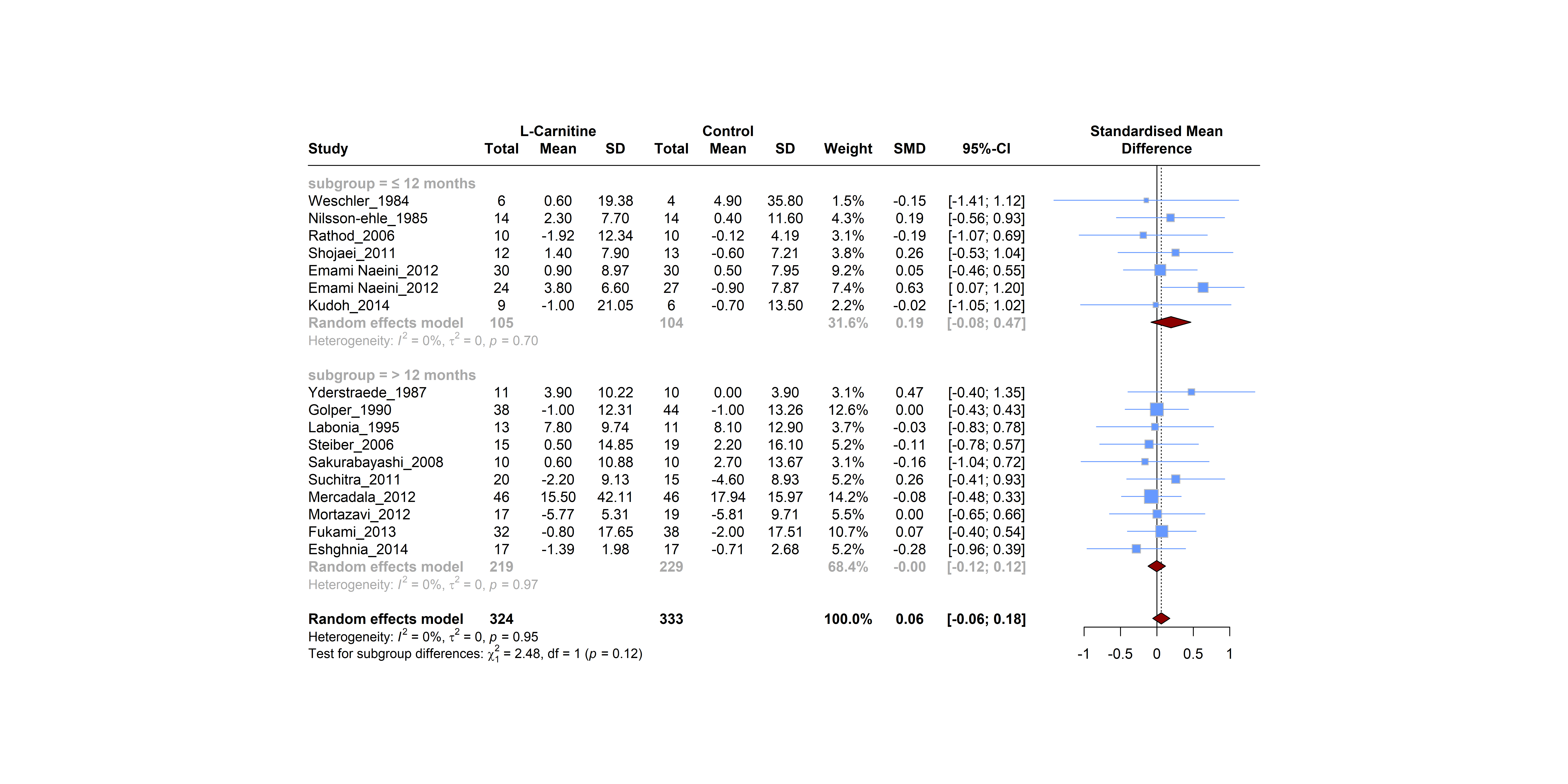  B: Duration |

**Figure S3.** Subgroup analysis based on the dosage (A) and treatment duration (B) for HDL

| 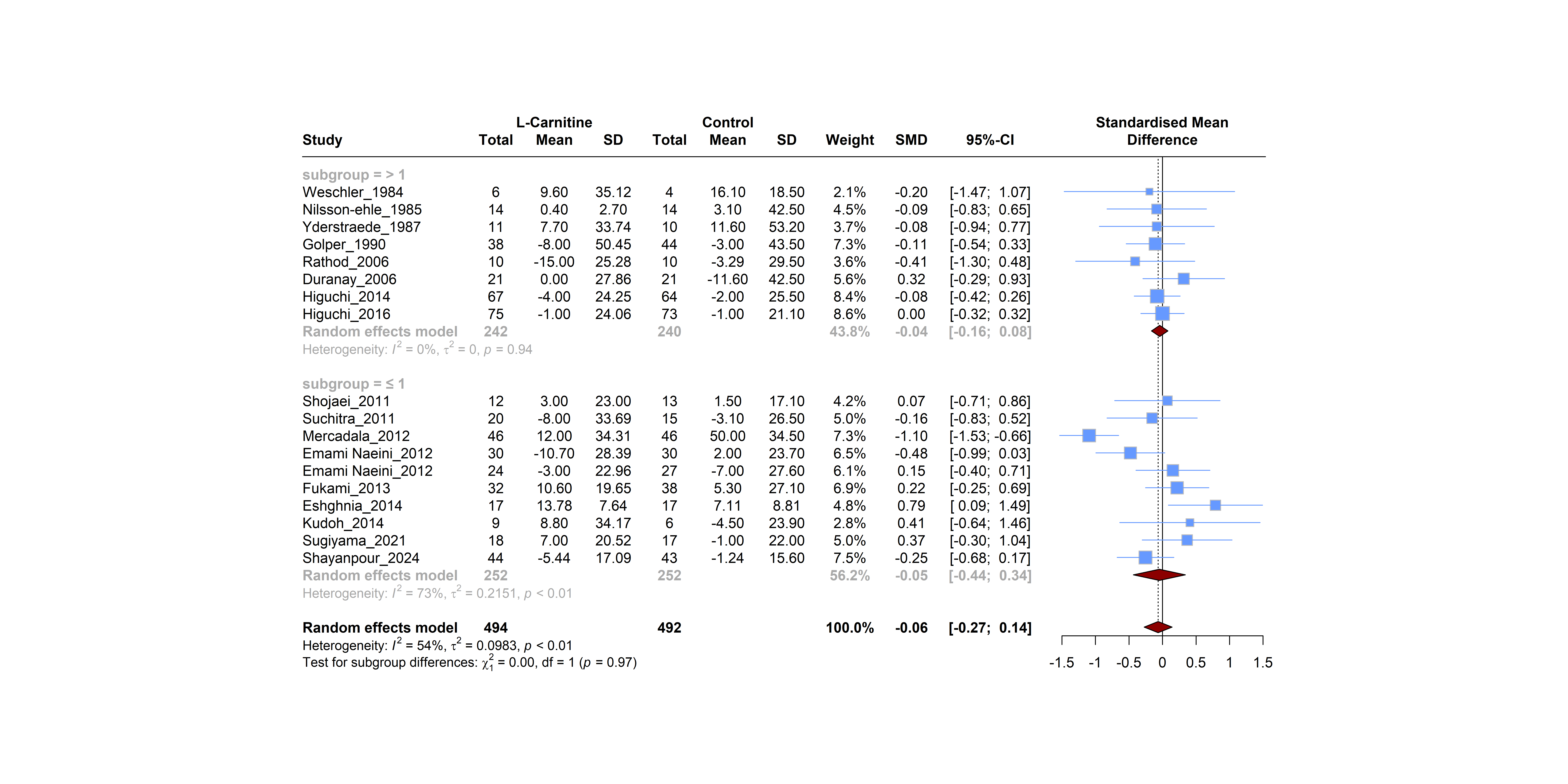  A: Dosage |
| --- |
| 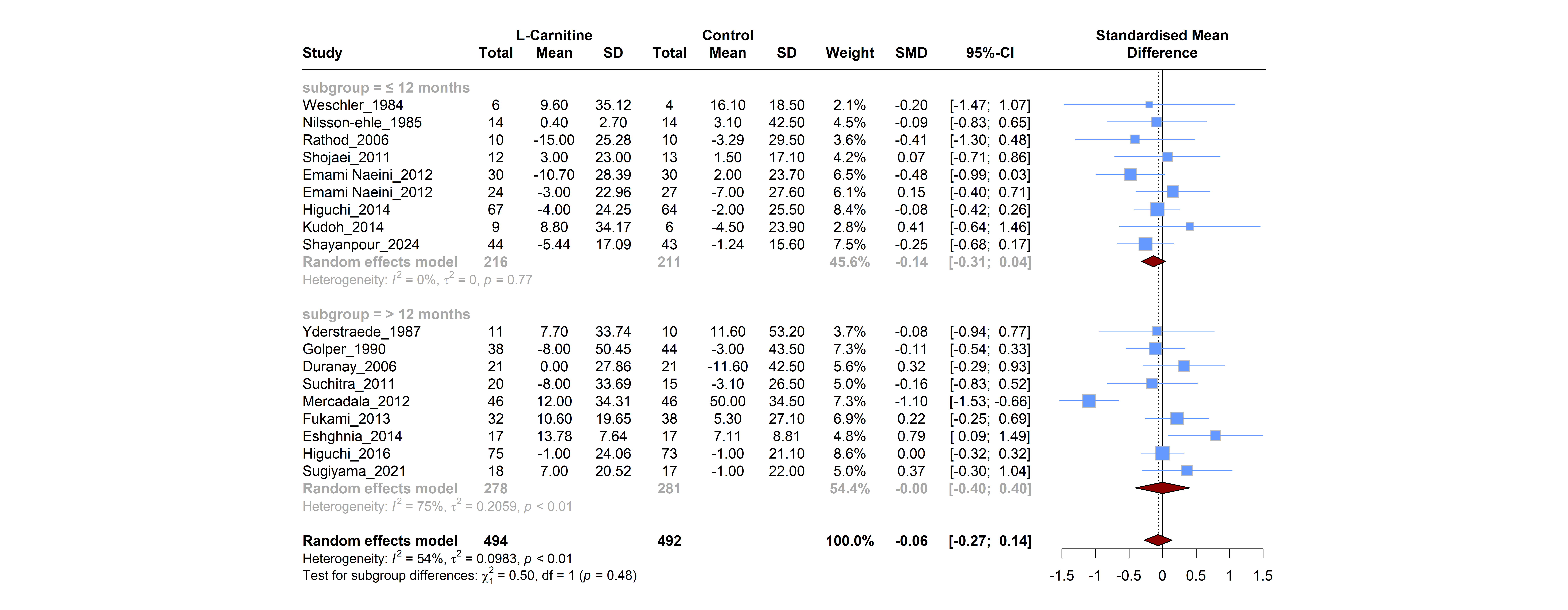  B: Duration |

**Figure S4.** Subgroup analysis based on the dosage (A) and treatment duration (B) for LDL

| 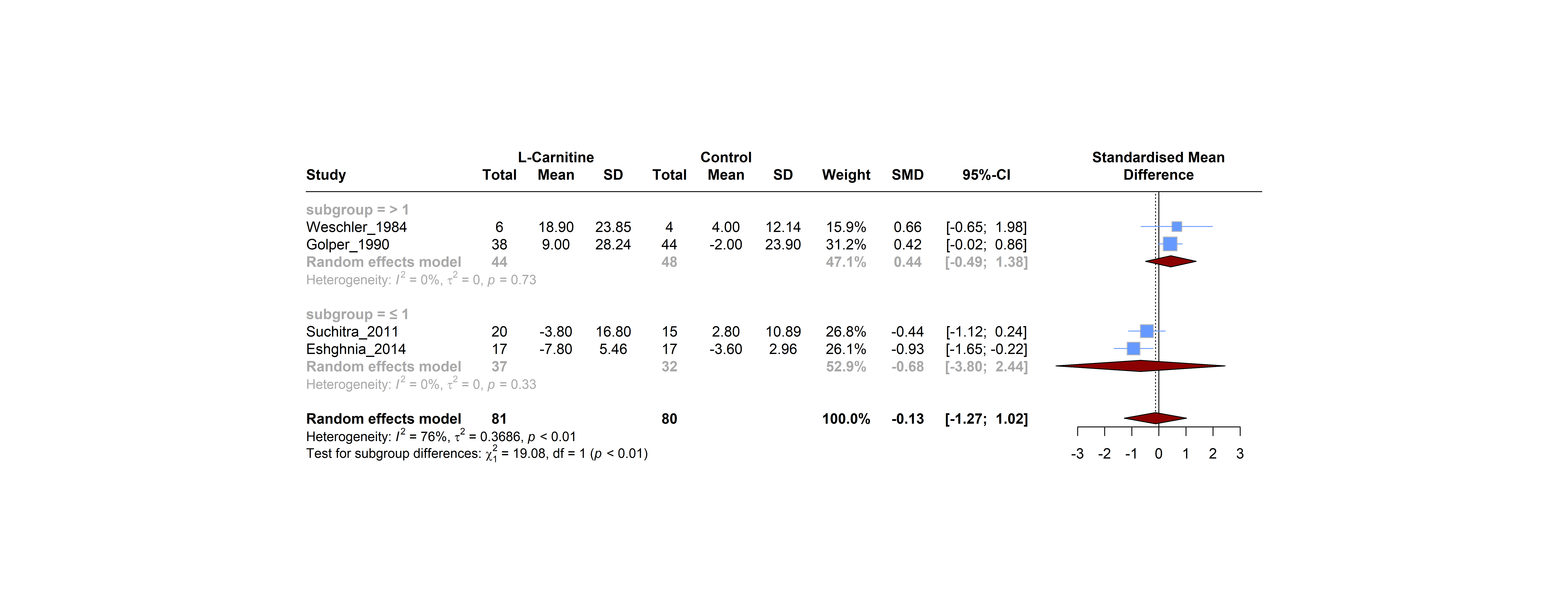  A: Dosage |
| --- |
| 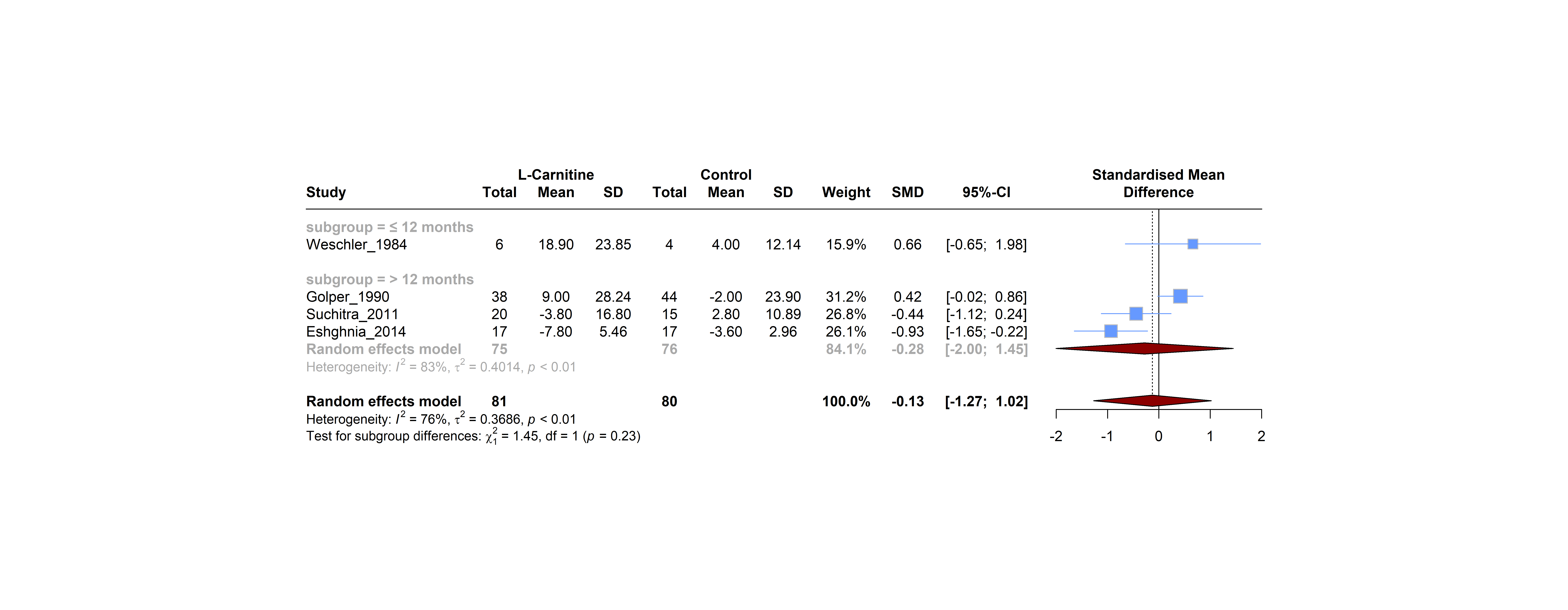  B: Duration |

**Figure S5.** Subgroup analysis based on the dosage (A) and treatment duration (B) for VLDL

| 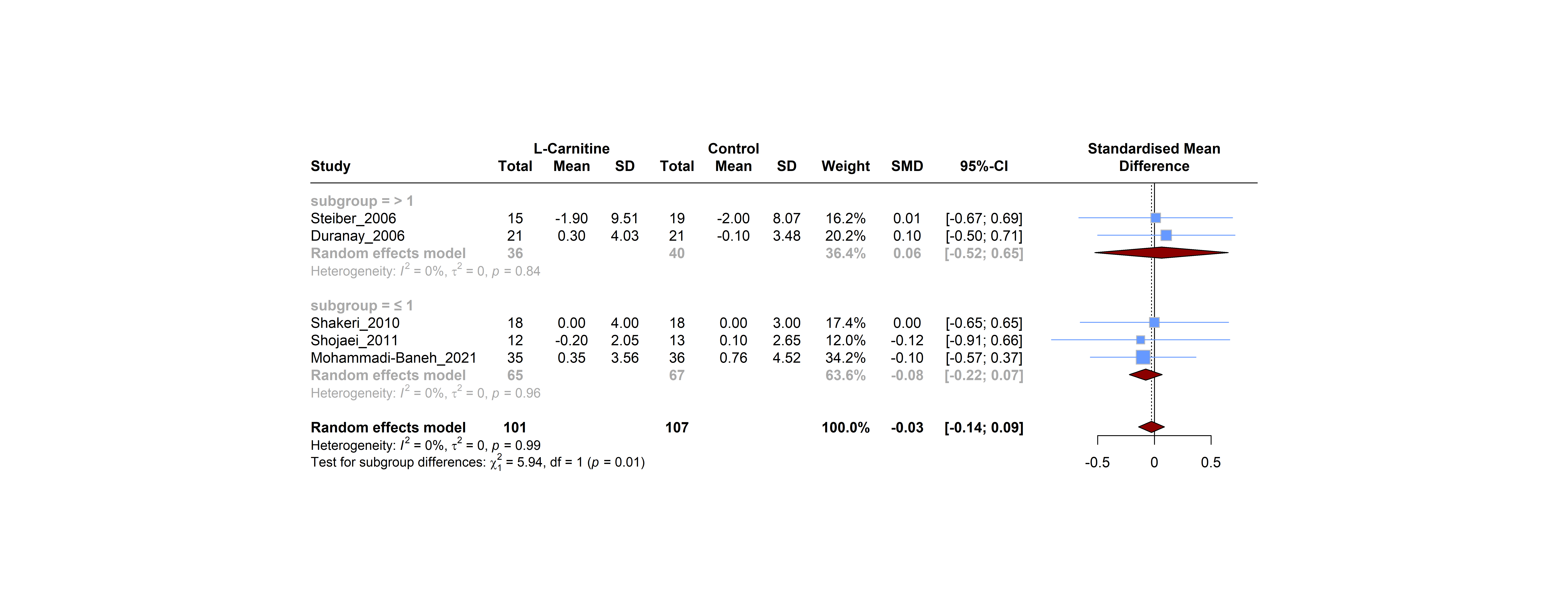  A: Dosage |
| --- |
| 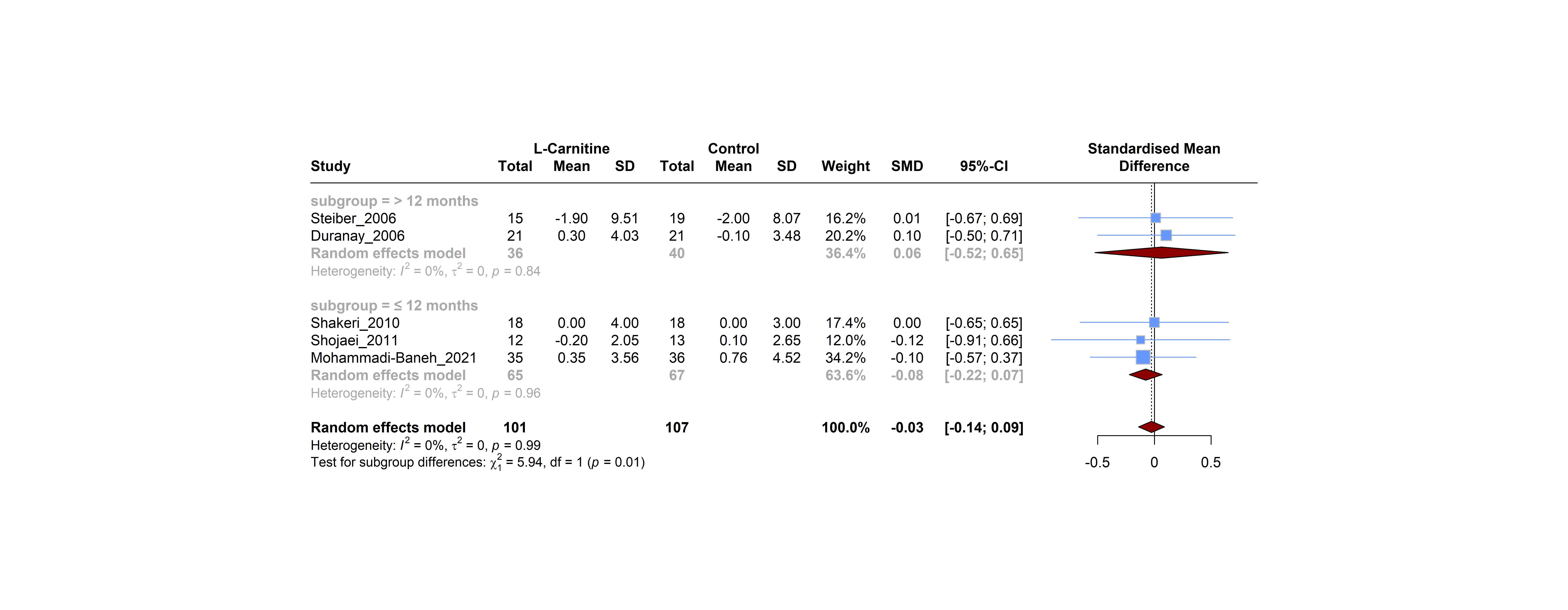  B: Duration |

**Figure S6.** Subgroup analysis based on the dosage (A) and treatment duration (B) for BMI

| 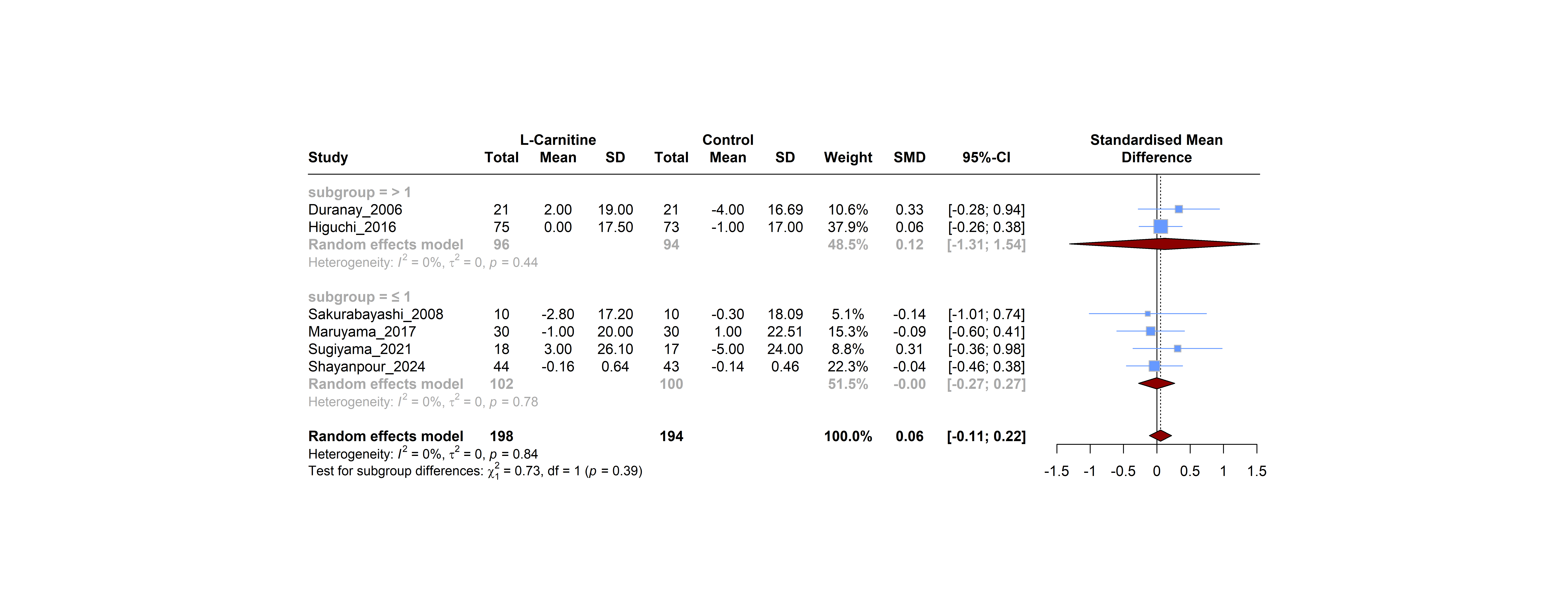  A: Dosage |
| --- |
| 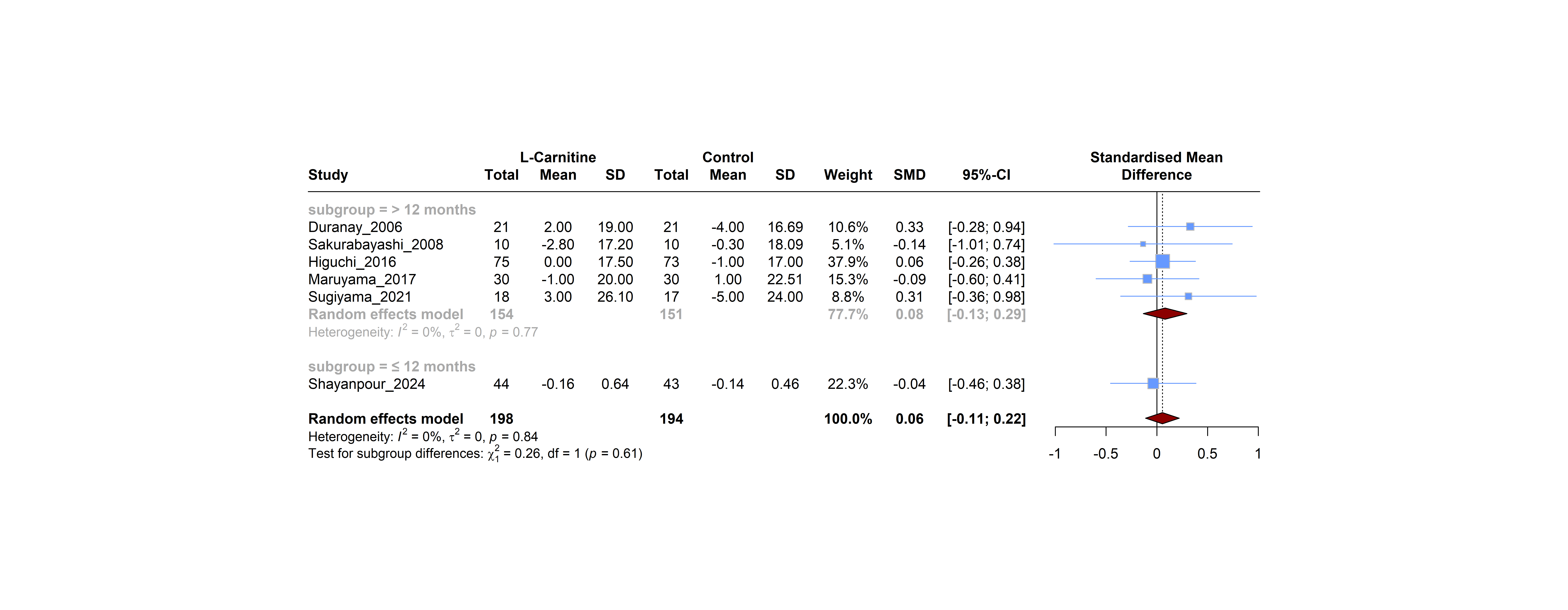  B: Duration |

**Figure S7.** Subgroup analysis based on the dosage (A) and treatment duration (B) for systolic blood pressure (BP)

| 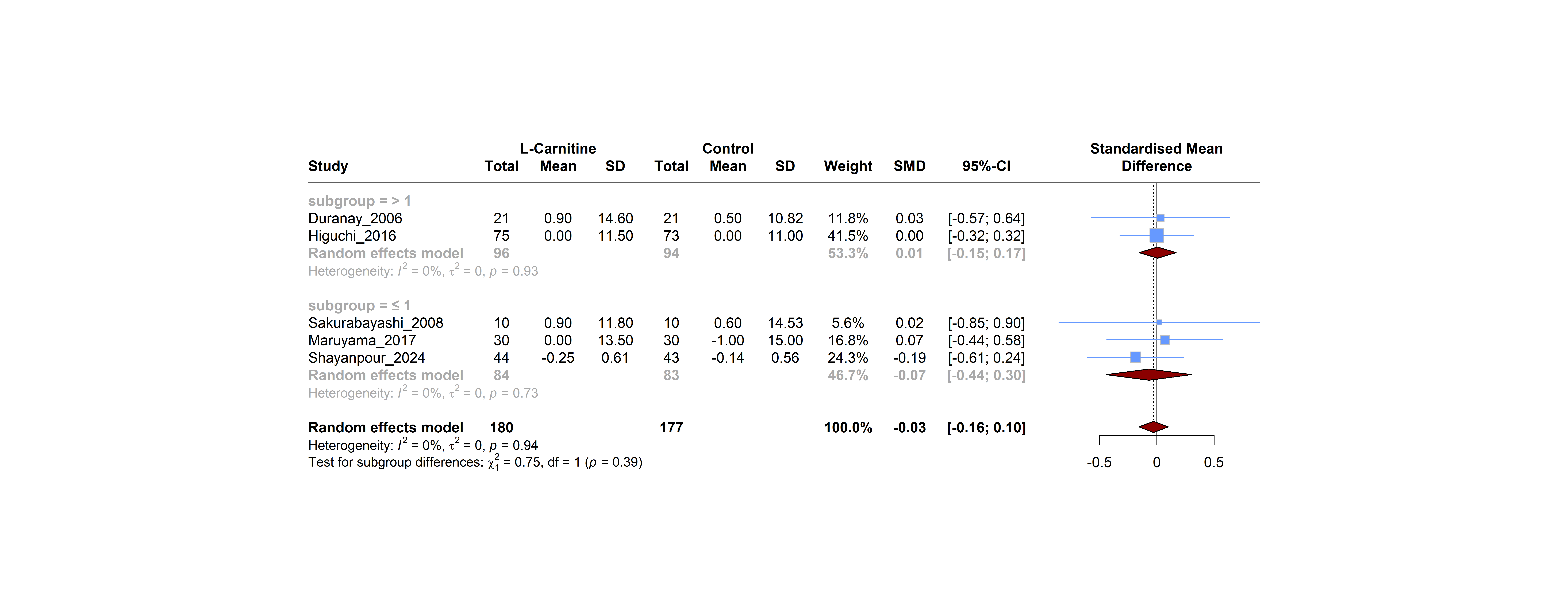  A: Dosage |
| --- |
| 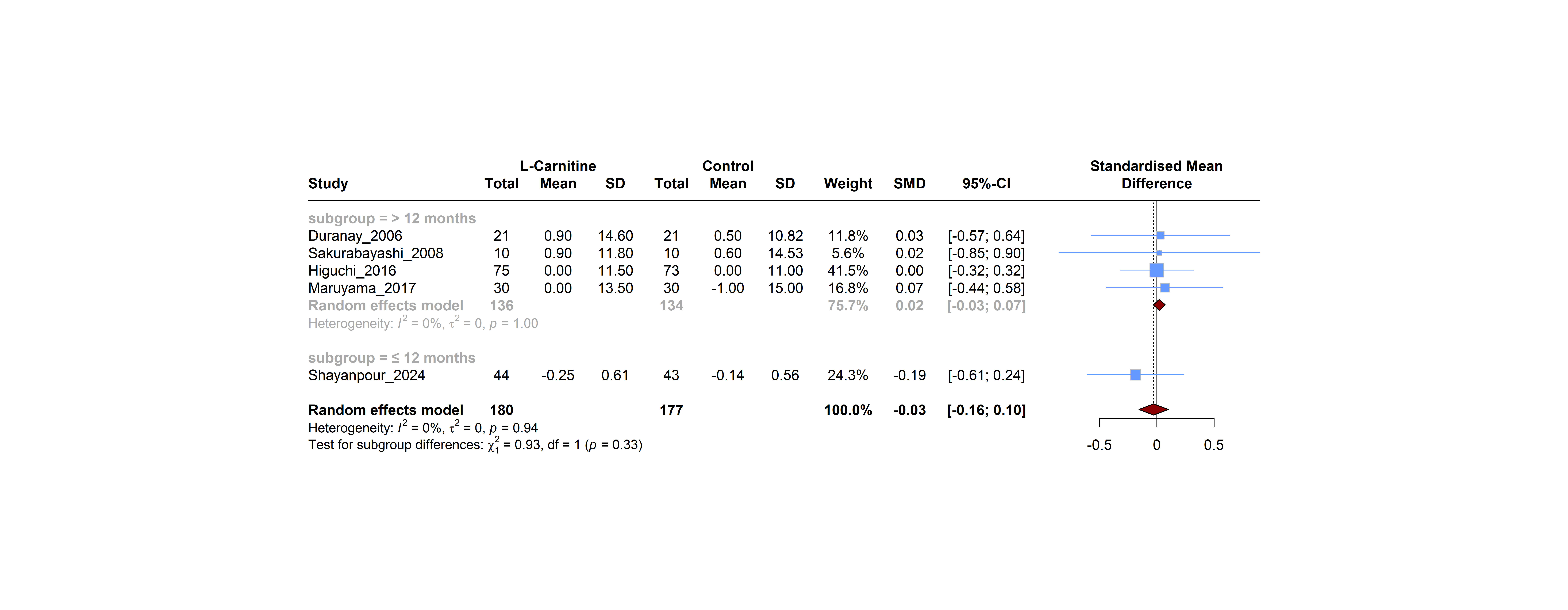  B: Duration |

**Figure S8.** Subgroup analysis based on the dosage (A) and treatment duration (B) for diastolic blood pressure (BP)

| **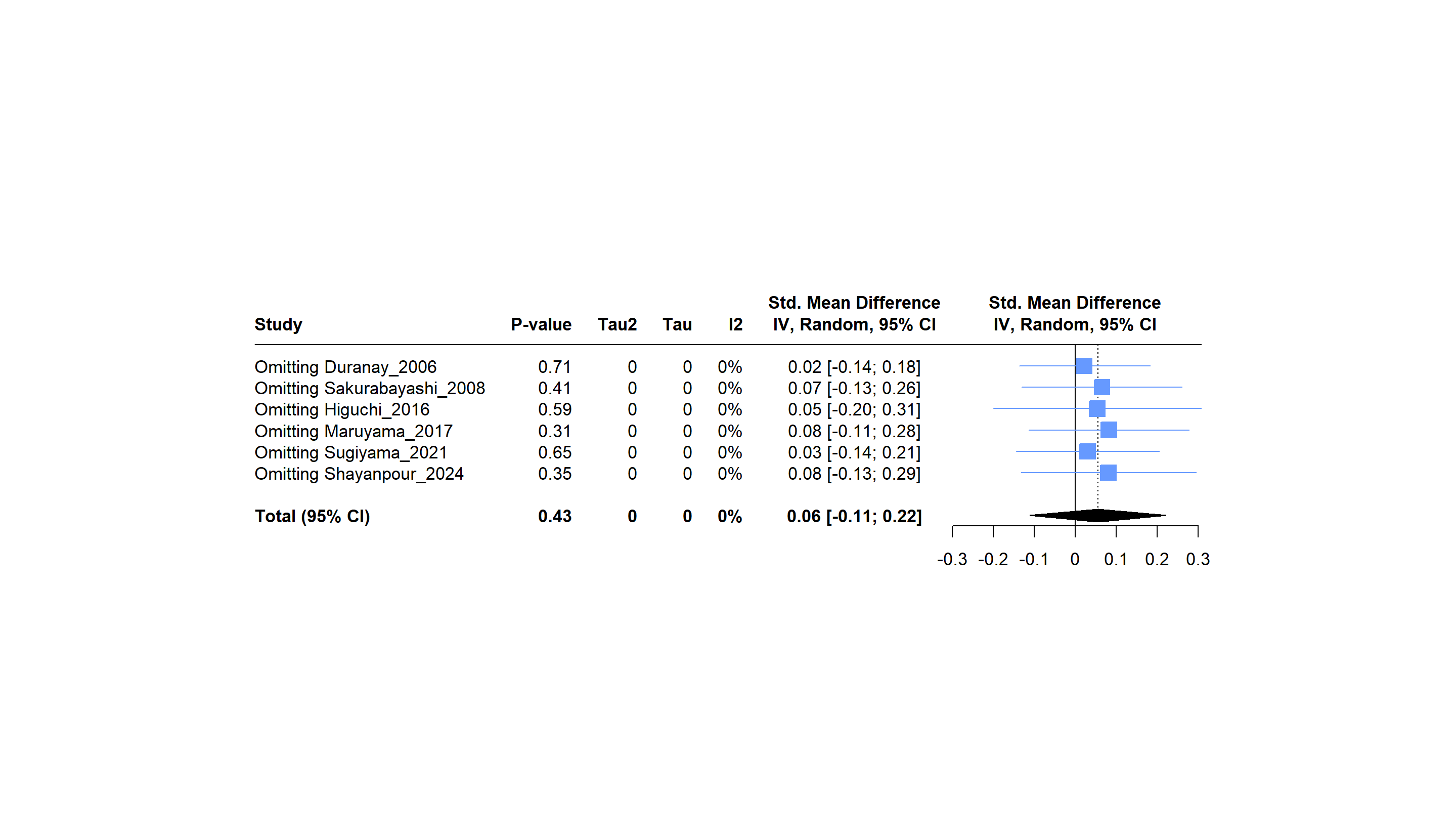**  **A: Systolic BP** |
| --- |
| 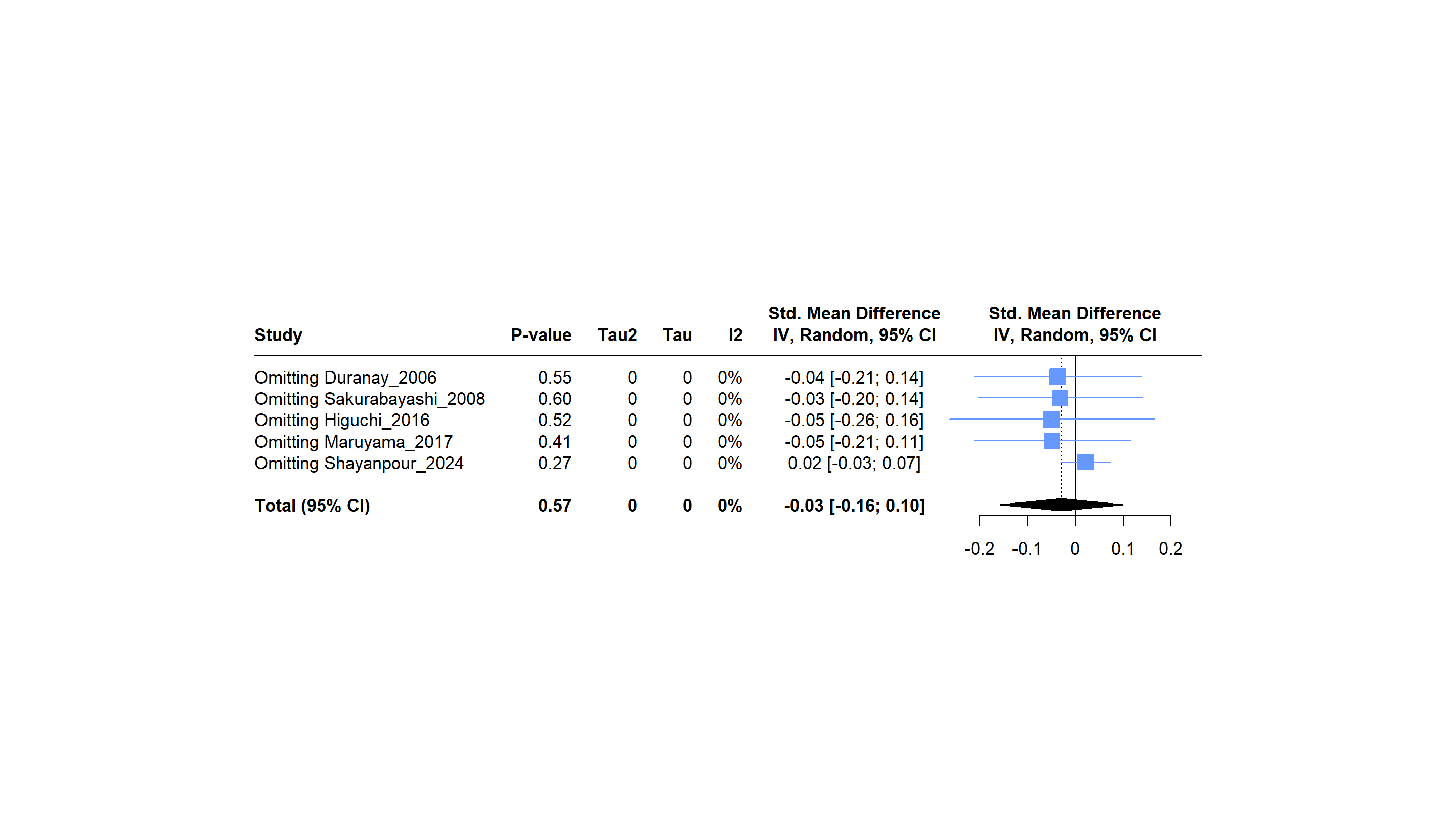  **B: Diastolic BP** |
| 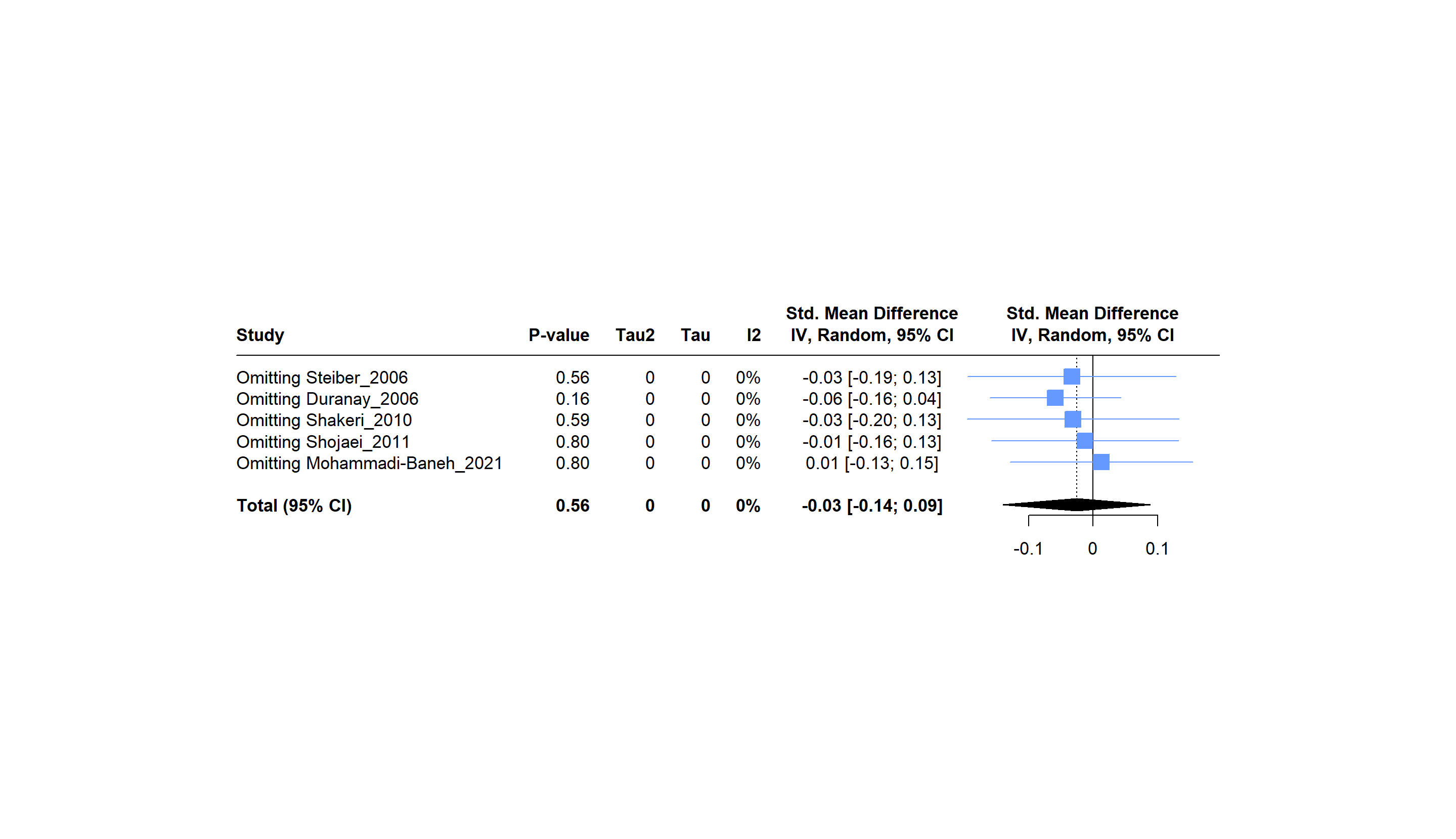  C: BMI |

**Figure S9.** Forest plot for sensitivity analysis based on the leave-one-out method of systolic (A) and diastolic (B) blood pressure (BP) and BMI (C) mean change in L-carnitine groups versus control groups


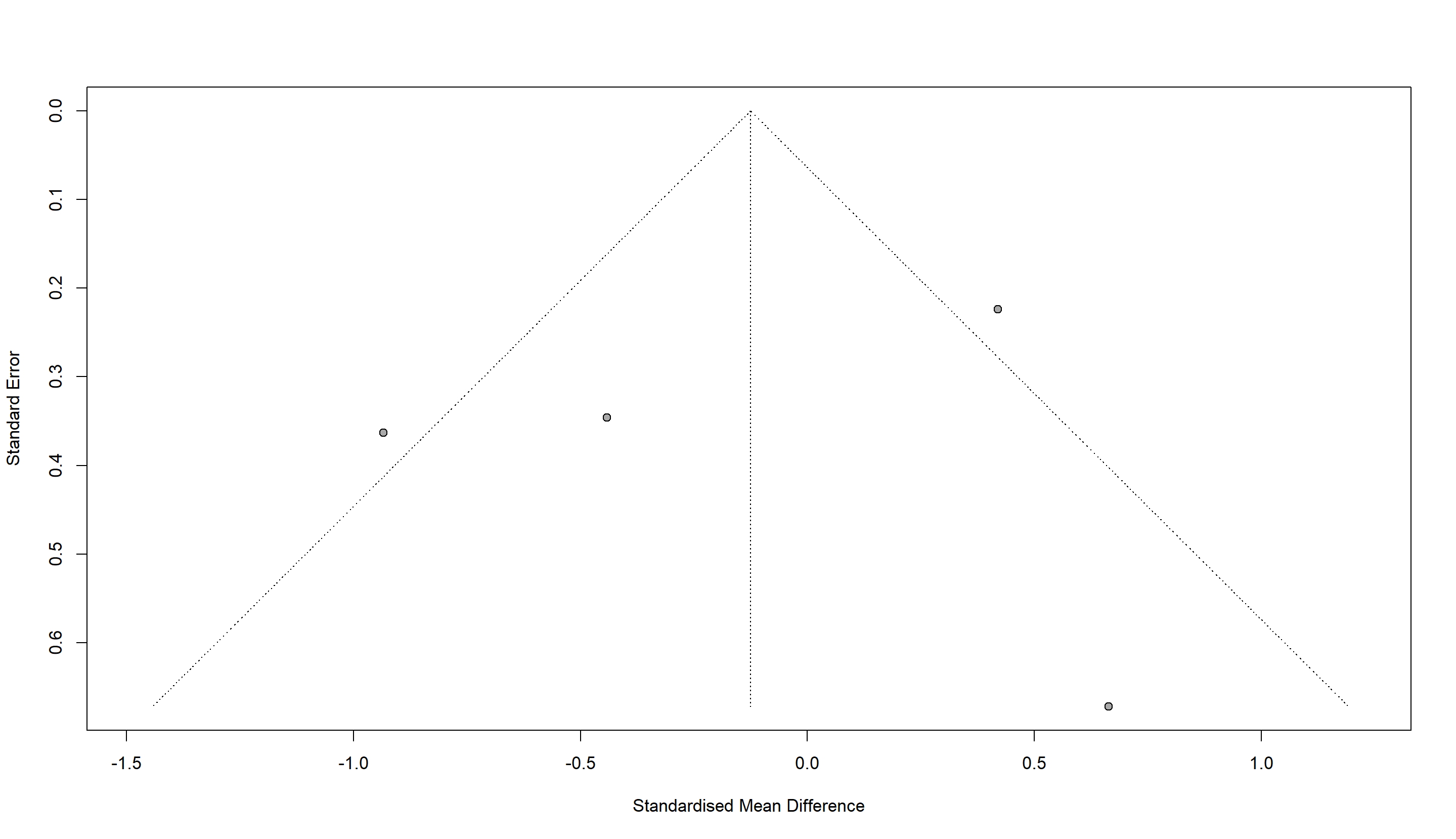


**Figure S10.** Funnel plot for risk of publication bias assessment based on trim and fill method of SMD of VLDL between L-carnitine groups versus control groups


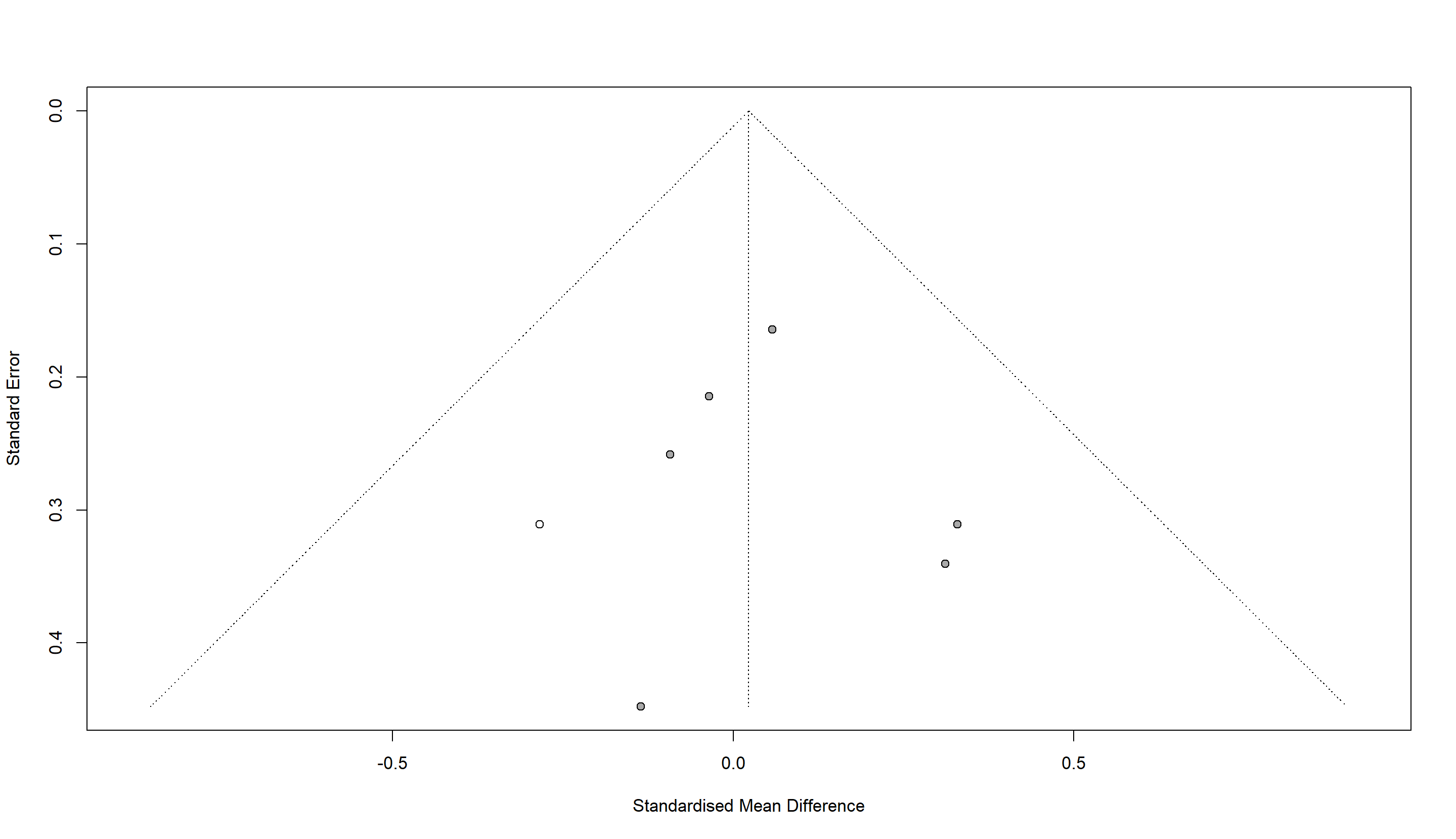


**Figure S11.** Funnel plot for risk of publication bias assessment based on trim and fill method of SMD of systolic BP between L-carnitine groups versus control groups.


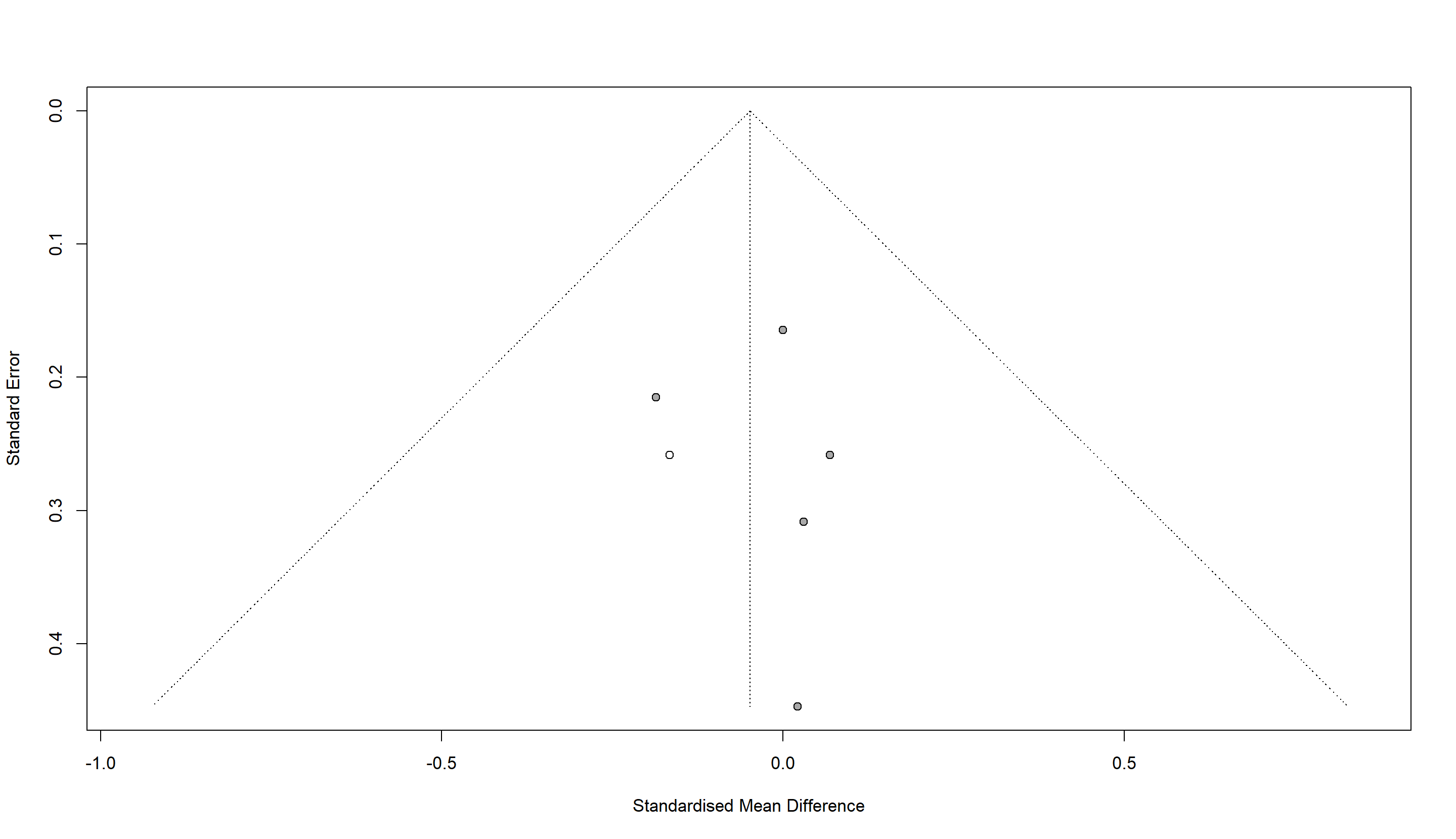


**Figure S12.** Funnel plot for risk of publication bias assessment based on trim and fill method of SMD of diastolic BP between L-carnitine groups versus control groups.


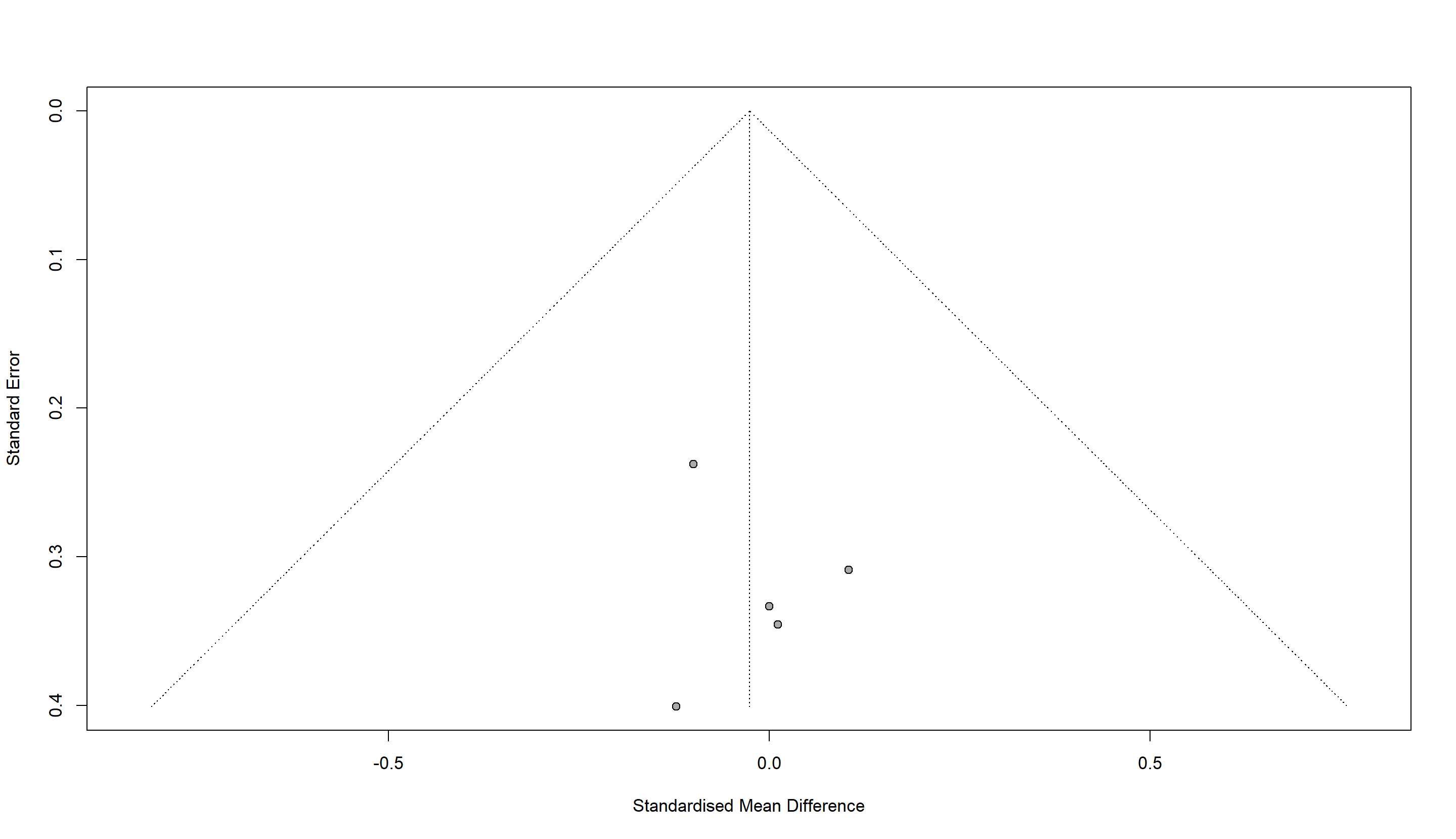


**Figure S13.** Funnel plot for risk of publication bias assessment based on trim and fill method of BMI between L-carnitine groups versus control groups.
